# Formative Research for Adapting the Cholera-Hospital-Based-Intervention-for-7-Days (CHoBI7) Water Treatment and Hygiene Mobile Health Program for Scalable Delivery in Rural Bangladesh

**DOI:** 10.3390/ijerph22020170

**Published:** 2025-01-26

**Authors:** Fatema Zohura, Tahmina Parvin, Kelly Endres, Elizabeth D. Thomas, Zakir Hossain, Kabir Hossain, Jahed Masud, Ismat Minhaj, Sawkat Sarwar, Jamie Perin, Mohammad Bahauddin, Md. Nazmul Islam, Sheikh Daud Adnan, Ahmed Al-Kabir, Abu S. G. Faruque, Christine Marie George

**Affiliations:** 1 Research, Training and Management International, Dhaka 1216, Bangladesh; 2 Department of International Health, Johns Hopkins Bloomberg School of Public Health, Baltimore, MD 21205, USA; 3 Bangladesh Ministry of Health and Family Welfare, Dhaka 1212, Bangladesh; 4 International Centre for Diarrhoeal Disease Research, icddr,b, Dhaka 1212, Bangladesh

**Keywords:** mHealth, behavior change, cholera and diarrhea, handwashing with soap, water treatment, water, sanitation, and hygiene, Bangladesh

## Abstract

The Cholera-Hospital-based-Intervention-for-7-Days (CHoBI7) mobile health (mHealth) program is a targeted water treatment and hygiene (WASH) program for the household members of diarrhea patients, initiated in the healthcare facility with a single in-person visit and reinforced through weekly voice and text messages for 3 months. A recent randomized controlled trial of the CHoBI7 mHealth program in urban Dhaka, Bangladesh, found that this intervention significantly increased WASH behaviors and reduced diarrhea prevalence. The objective of this present study was to conduct formative research using an implementation science framework to adapt the CHoBI7 mHealth program for scalable implementation in rural Bangladesh, and to promote construction of self-made handwashing stations (CHoBI7 Scale-up program). We conducted a 3-month multi-phase pilot with 275 recipients and 25 semi-structured interviews, 10 intervention planning workshops, and 2 focus group discussions with intervention recipients and program implementers. High appropriateness, acceptability, and adoption of the CHoBI7 Scale-up program was observed, with most recipients constructing self-made handwashing stations (90%) and chlorinating drinking water (63%) and 50% of participants observed handwashing with soap in the final pilot phase. At the recipient level, facilitators included weekly voice and text messages with videos on handwashing station construction, which served as reminders for the promoted water treatment and hand hygiene behaviors. Barriers included perceptions that self-made iron filters commonly used in households also removed microbial contamination from water and therefore chlorine treatment was not needed, and mobile messages not always being shared among household members. At the implementer level, facilitators for program implementation included follow-up phone calls to household members not present at the healthcare facility at the time of intervention delivery, and the promotion of multiple self-made handwashing station designs. Barriers included high patient volume in healthcare facilities, as well as the high iron in groundwater in the area that reduced chlorination effectiveness. These findings provide valuable evidence for adapting the CHoBI7 mHealth program for a rural setting, with a lower-cost, scalable design, and demonstrated the important role of formative research for tailoring WASH programs to new contexts.

## 1. Introduction

Diarrhea is a leading cause of mortality among children under 5 years old worldwide, resulting in approximately 445,000 deaths annually [1]. Water, sanitation, and hygiene (WASH) programs promoting water treatment and handwashing with soap can significantly reduce diarrheal disease incidence among young children [2,3,4]. However, community-based WASH programs are often expensive and difficult to implement effectively in resource-constrained settings [5,6]. Furthermore, challenges with scalability of the intervention program and sustainability of promoted WASH behaviors have been major barriers to the successful implementation of WASH programs globally [5]. Effective, scalable programs are urgently needed to reduce diarrheal diseases around the world and promote sustained WASH behavior change.

Targeting WASH programs to populations at high risk for diarrheal diseases presents a cost-effective approach for delivering WASH to populations that will likely benefit the most [7]. Previous studies in Bangladesh have demonstrated that household members of diarrhea patients are at much higher risk of diarrheal diseases than the general population (100 times higher risk for cholera) during the 7 days after the presentation of the index diarrhea patient in the household at a healthcare facility [8,9]. This increased risk is likely from household members of diarrhea patients sharing the same contaminated drinking water sources and a lack of handwashing with soap, both within health facilities and in the household [8,9,10]. Despite the higher risk of diarrheal diseases for the household members of diarrhea patients, there are limited interventions that target this population [7,11].

The Cholera-Hospital-based-Intervention-for-7-Days (CHoBI7) program is a safe drinking water and handwashing with soap intervention, targeted to the household members of diarrhea patients. The CHoBI7 program is healthcare facility-initiated, with an initial pictorial WASH module (flipbook) delivered by a health promoter bedside to diarrhea patients and their accompanying household members in the healthcare facility during the time of treatment. The importance of handwashing with soap and safe drinking water treatment and storage for diarrhea prevention was then reinforced in the initial version of this program through home visits during the 7-day high-risk period after the diarrhea patient is admitted to the healthcare facility. A diarrhea prevention package was also provided, which included a handwashing station (bucket with tap and basin underneath), soapy water bottle (water and detergent powder in a plastic bottle), drinking water vessel with lid and tap, and chlorine tablets. In the initial randomized controlled trial (RCT) of the CHoBI7 program in urban Dhaka, Bangladesh, the intervention significantly reduced symptomatic cholera infections among the household members of cholera patients [12].

The findings from this first RCT of CHoBI7 led to a partnership with the Bangladesh Ministry of Health and Family Welfare to develop a scalable approach to deliver the intervention in Bangladesh. The first modification made to the original CHoBI7 program was to remove home visits, which would be a costly approach to scale in urban settings in Bangladesh that have limited community health worker infrastructure. In place of home visits, a mobile health component was developed to reinforce the information and recommendations from the WASH module delivered in the healthcare facility. The intervention was designed through community-centered, formative research, which included semi-structured interviews and a pilot study [7,13]. Mobile health (mHealth) presents an innovative, scalable intervention approach, where mobile messages can be sent to a large number of households at minimal cost (a total of USD 2 for 12 months of weekly voice messages), with demonstrated impacts on disease prevention practices [7,14,15,16,17]. In the second version of the CHoBI7 mHealth program, weekly voice and text messages were sent to the mobile phones of diarrhea patient households by a physician at a local diarrhea hospital, promoting handwashing with soap, water treatment, and safe water storage. The second modification made to the original CHoBI7 program for the mHealth iteration was to broaden the program scope from focusing on cholera patients to include diarrhea patients of all etiologies [7]. The same diarrhea prevention package from the original CHoBI7 program was also provided. In the RCT of the CHoBI7 mHealth program in urban Dhaka, Bangladesh, this more scalable approach resulted in significantly lower diarrhea prevalence and stunting 12 months after initial intervention delivery [13,18].

We are currently partnering with the Bangladesh Ministry of Health and Family Welfare to implement a scaling plan of the CHoBI7 mHealth program in Bangladesh over the next 5 years. In this paper, we present the community-centered, formative research conducted to (1) adapt the CHoBI7 mHealth program for delivery in rural areas in Bangladesh (all previous work has been in an urban setting) and (2) modify the diarrhea prevention package to include only a soapy water bottle and chlorine tablets (a lower-cost and more scalable package than also providing a handwashing station and drinking water vessel).

## 2. Materials and Methods

### 2.1. Study Overview

The objective of this study was to adapt the CHoBI7 mHealth program for a rural setting in Bangladesh and test the delivery of a modified diarrhea prevention package (soapy water bottle and chlorine tablets only). The adapted intervention is referred to here as the CHoBI7 Scale-up program. Adaptations were made through community-centered, formative research conducted from July 2022 to May 2024 in a rural area of Manikganj district in Bangladesh. Formative research activities were conducted as part of a pilot study, which included semi-structured interviews (SSI), focus group discussions (FGD), unannounced spot checks, and structured observations in the household to assess WASH conditions and behaviors. Formative research was community-centered; SSIs and FGDs engaged participants in a discussion on facilitators and barriers to the promoted WASH behaviors and recommendations on intervention refinement, which then informed revisions to intervention content and delivery. Community members also served as promoters for intervention delivery.

### 2.2. Study Eligibility Criteria and Enrollment Activities

For the pilot study, we recruited diarrhea patients from the Manikganj district hospital (tertiary level care) and five sub-district health complexes (secondary level care) (Table 1). Index diarrhea patient screening eligibility was the following: (1) had three or more loose stools in the past 24 h; (2) resided in their home for the three nights prior to hospitalization; (3) had no running water inside their home; (4) had a child under five years of age in the household (given that children < 5 years are at high risk for diarrhea); and (5) had a working mobile phone in the household. Household members of the diarrhea patient were eligible if (1) they shared the same cooking pot and resided in the same home with the diarrhea patient for the last three days and (2) they planned to reside with the diarrhea patient for the next 3 months. Recruitment of diarrhea patients occurred from October 2022 to December 2023. Pilot participants were selected using convenience sampling of patients that were admitted to healthcare facilities during the dates of the pilot study and who met the screening eligibility criteria. Patients were not excluded on any characteristics beyond our screening eligibility criteria. After enrolling the index diarrhea patient, their household members were enrolled either in the healthcare facility or at their home. The demographic characteristics of households at enrollment are presented in Table 1. 

### 2.3. CHoBI7 mHealth Scale-Up Program Delivery

**Figure 1:**
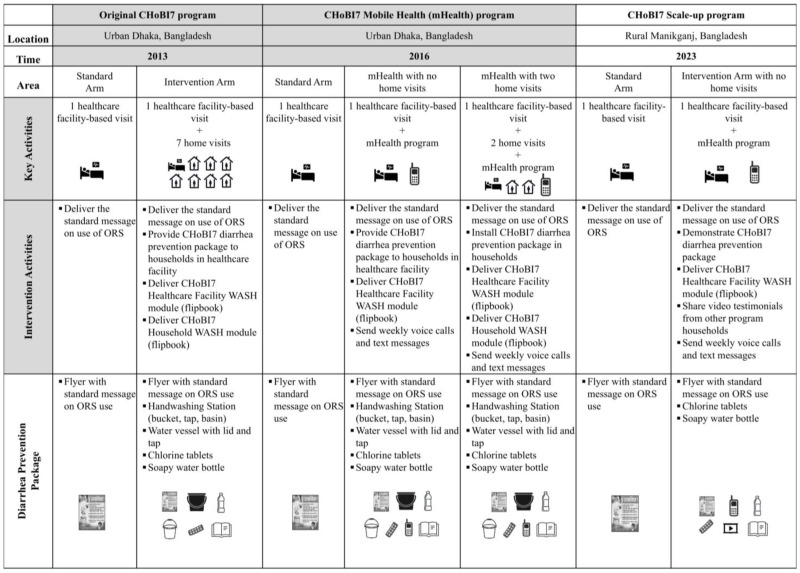
Overview of CHoBI7 program activities developed from 2013 to 2024.

The starting point for the pilot study intervention was the CHoBI7 mHealth program delivered in our previous RCT in Dhaka, Bangladesh [18]. In Figure 1, we provide an overview of the versions of the CHoBI7 program delivered over the past 11 years, including key activities and intervention components.

For this present study, the CHoBI7 mHealth program was adapted to the CHoBI7 Scale-up program as described in the results section. At the start of the pilot study, the CHoBI7 Scale-up program had the following components: (1) a healthcare facility visit by a health promoter to deliver the WASH module during the time of treatment for the index diarrhea patient; (2) a modified diarrhea prevention package with only a soapy water bottle and chlorine tablets (60 tablets) and sticker and cue cards on the promoted WASH behaviors; and (3) weekly voice, interactive voice response (IVR), and text messages (using the EngageSPARK mobile platform [19]), sent for 3 months to the mobile phones of diarrhea patient households to reinforce the intervention content provided in the healthcare facility. All health promoters resided in the local community. Videos of actual community members explaining the promoted WASH behaviors were added to the intervention before the pilot. The CHoBI7 Scale-up program promotes five behaviors: (1) constructing a handwashing station, defined as a place where water and soap are available for handwashing; (2) handwashing with soap at food- and stool-related events; (3) safe drinking water storage in a water vessel with a lid; (4) drinking water treatment using chlorine tablets during the 7-day high-risk period after diarrhea patient admission; and (5) drinking water treatment by heating of the household water until it reaches a rolling boil after the 7-day high-risk period (or when chlorine tablets are no longer available in their household).

Pilot participants received either the CHoBI7 Scale-up program (intervention arm) or the 5 min standard message provided in public healthcare facilities to diarrhea patients during discharge from the healthcare facility on the use of oral rehydration solution (ORS) (provided by a health promoter and on a flyer) (standard-message arm). Study-arm assignment was based on the day and the ward to which diarrhea patients were admitted during their healthcare facility stay to reduce the likelihood that standard-message-arm participants would have exposure to the intervention. In this study, there were five phases of iterative intervention development and pilot testing. Supplementary Figure S1 provides an overview of the iterative process of pilot testing over the five phases.

### 2.4. Pilot Quantitative Data Collection Methods

A five-hour structured observation was conducted at Day-7, 1 month, and 3 months after enrollment in all available pilot households to observe handwashing with soap at food- and stool-related events, following our previously published methods [18]. Unannounced spot checks were also conducted in all households at 1 week, 1 month, and 3 months after enrollment to assess the presence of free available chlorine in stored household drinking water, also using published methods [20,21]. This data was compiled into an intervention uptake report for the research intervention and evaluation teams to review and discuss during intervention planning workshops.

### 2.5. Pilot Qualitative Data Collection Methods

From December 2022 to May 2024, a total of 25 SSIs and 2 FGDs were conducted to explore intervention implementers’ and recipients’ (pilot participants) experiences with the intervention, including successes and challenges with the promoted WASH behaviors and recommendations on how to improve the implementation and content of the CHoBI7 Scale-up program (Supplementary Table S1). The SSI and FGD guides for pilot participants included questions on the experiences pilot participants had with the following components of the intervention: healthcare facility delivery of the WASH module, water treatment with chlorine tablets, construction of a handwashing station, using a safe water storage vessel, using a soapy water bottle, cue cards on WASH behaviors, and mobile messages (voice and text messages). There was one FGD conducted with women and another with men. The four intervention implementers were interviewed at the end of the pilot, and their interview guide was based on the following implementation outcomes: acceptability, adoption, appropriateness, cost, fidelity, feasibility, penetration, sustainability, and scalability [22]. No FGDs or interviews were conducted with phase 3 participants due to political unrest at the time. 

Interview and FGD participants were purposefully identified based on intervention uptake reports and observations by research staff during household visits. For example, when an intervention uptake report showed low uptake of handwashing with soap by structured observation, we then purposefully selected households with low observed handwashing to interview to help us understand the barriers individuals faced with this behavior. All interviews and FGDs were conducted by members of the CHoBI7 evaluation team trained in qualitative data collection, who administered guides adapted from versions employed in previous CHoBI7 studies [7,13]. In addition, after the Month 3 structured observation and spot check visit, research staff conducted additional “field note” unannounced household visits to observe WASH conditions in the households and to ask households about the challenges they faced during the study period. Field notes were compiled during these visits, which were separate from the SSIs.

### 2.6. Intervention Planning Workshops

Ten intervention planning workshops were conducted throughout the pilot study to adapt protocols for intervention delivery based on findings from each phase of the pilot. Intervention adaptations included revising the content of (1) the WASH module, (2) demonstrations of the promoted behaviors, (3) testimonial videos, (4) cue cards, and (5) mobile messages. Each intervention workshop included the project intervention coordinator, evaluation activity coordinator, health promoters, a research officer, and research assistants. Workshops were conducted as either a half-day or full-day session at the project office. Qualitative and quantitative findings from the pilot were discussed as a team, including the findings from structured observations, unannounced spot checks, SSIs, FGDs, and field notes from observations of intervention delivery. A participatory approach was used to decide on intervention modifications based on the consensus of the entire research team.

### 2.7. Data Analysis

All interviews and FGDs were conducted in Bangla and audio-recorded. Based on the questions in the interview guides and emerging themes, all recordings were reviewed to develop an analytical summary template. The analysis summary was completed for all FGDs and SSIs. All related quotes were transcribed verbatim in Bangla and then were translated into English. Field notes further supplemented the analysis questionnaires. The recordings, analysis questionnaires, and the translation of the quotes were synthesized in a matrix according to the levels and factors of the Integrated Behavioral Model for Water, Sanitation, and Hygiene (IBM-WASH) to facilitate interpretation of findings and identify intervention components that would require modification [23]. An implementation science framework was also applied to organize study findings from SSIs by implementation outcomes: adoption, acceptability, appropriateness, cost, feasibility, fidelity, penetration, and sustainability [22].

### 2.8. Ethical Approval

This study was approved by the National Research Ethics Committee (NREC) of the Bangladesh Medical Research Council (BMRC) (Reference No. BMRC/NREC/2019-2022/119), the NGO Bureau (Protocol PR-03.07.2666.664.68.027.22.194), and the Johns Hopkins Bloomberg School of Public Health (Protocol IRB NO. 000015070). This study also received permission from the Directorate General of Health Services and the directors of the district and sub-district healthcare facilities in Manikganj, Bangladesh. All study participants provided written informed consent, or parental permission was obtained from a guardian. Measures taken to ensure participant protection included collecting and storing all participant data using encrypted computers, files, databases, and servers and storing all consent forms and recording devices in a locked file cabinet. Participant phone numbers were stored on an encrypted server, and mHealth messages were sent using an encrypted platform.

## 3. Results

### 3.1. CHoBI7 Program Adaptations for Scalable Delivery in a Rural Setting

Intervention adaptations for the CHoBI7 Scale-up program occurred before and throughout the five pilot phases. The adaptations explored below are organized into three general categories: changes to the guidance provided on construction of self-made handwashing stations (Section 3.1.1, Section 3.1.2, Section 3.1.3, Section 3.1.4, Section 3.1.5 and Section 3.1.6), changes to the mHealth components (Section 3.1.7, Section 3.1.8, Section 3.1.9, Section 3.1.10, Section 3.1.11, Section 3.1.12 and Section 3.1.13), and changes related to water treatment guidance (Section 3.1.11, Section 3.1.12, Section 3.1.13 and Section 3.1.14). A timeline overview of program adaptations is provided in Supplementary Table S2.

#### 3.1.1. Initial Development of Modified Healthcare Facility CHoBI7 WASH Module (Flipbook)

The initial changes to the CHoBI7 flipbook (WASH module) prior to the start of the pilot included updating the images to reflect water sources and water storage containers used in rural Bangladesh (rather than an urban setting). Instructions were also added on how to construct a handwashing station using a bucket or plastic drum with a tap, and a vignette of a family was included, named “50 taka can buy happiness”. The vignette described two families enrolled in the CHoBI7 Scale-up program. The first family had a household member that was admitted to a healthcare facility for diarrhea who bought a tap for 50 taka (USD 0.50) and constructed their own handwashing station and subsequently remained diarrhea-free during the 7-day high-risk period. This family was compared to another family who did not prepare the handwashing station and whose young child became sick with diarrhea, and the family had to return to the healthcare facility during the 7-day high-risk period. After this vignette, diarrhea patient households were provided a sheet with a list of shop addresses that sold taps for 50 taka in their area. For water treatment and safe water storage, a flipbook page was included on how to correctly treat drinking water with chlorine tablets (e.g., correct dosing) using locally available water storage vessels. One demonstration session was also conducted in the healthcare facility based on this page. A flipbook page was also added to the WASH module explaining that because latrines and tubewells are often within a few meters of each other in the rural setting, there is a high potential for contamination of drinking water collected from the tubewell.

#### 3.1.2. Videos on WASH Behaviors

Prior to the start of the pilot, a three-part video on the promoted WASH behaviors was created. The first part was a testimonial video of a father who received the CHoBI7 intervention while his young child was at a healthcare facility for diarrhea. He explained the importance of the intervention for the health of his family. During the video, we observe his journey as he goes to a local shop to buy a tap to make a handwashing station for his family. He is then shown using a hot pipe to put a hole in a bucket so that he can add a tap to his handwashing station. He also demonstrates how to prepare soapy water. The final two parts of the video are focused on the children in the household. One video is of a young boy that became sick with diarrhea and was not able to play with his friends. He tells the viewers of the video that after he received the intervention, he followed the recommended WASH behaviors and he became healthy again and was able to play with his friends. The final video is of a group of children reciting a nursery rhyme, developed by the study team, that explains the key times to wash hands with soap and the importance of drinking chlorine-treated water.

#### 3.1.3. Demonstrations of Handwashing Station Designs

**Figure 2:**
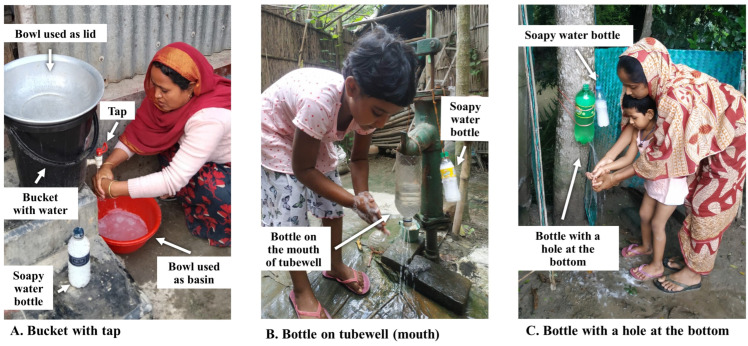
Three handwashing station designs developed for CHoBI7.

At the start of the pilot study, we set up a handwashing station “demonstration station” in each study healthcare facility. The demonstration handwashing station design was a “bucket with tap” (Figure 2A). During delivery of the WASH module (flipbook) in the healthcare facility, pilot participants were shown how to make a hole in a plastic bucket to insert a tap, and then practiced how to add a tap to a demonstration bucket. Each participant present during the module practiced adding a tap to the demonstration bucket. During the demonstration, participants also practiced washing their hands using the handwashing station under the guidance of the health promoter, with soapy water that they prepared themselves. From qualitative findings, this component of the intervention was well received, and some households modified our recommended design based on the materials they had available in their home. Examples of handwashing stations created by program participants are shown in Supplementary Figure S2.

“*I was not taught how to put a faucet [tap] on a bottle, I made it myself… [I] made it with a 5 L oil bottle... If you want to buy a bucket, you have to go to the market, it will cost money... So, I had this idea [adding a tap on a bottle]… I made [the handwashing station] with what I have at home.*”

(SSI, Female)

#### 3.1.4. Introduction of a New Design of a Handwashing Station on a Tubewell

During interviews, some pilot participants mentioned that they did not have an extra bucket or drum in their home and could not afford to buy one to make the “bucket with tap” handwashing station. Additionally, some participants mentioned that their children played with the wastewater from the “bucket with tap” design, which made it challenging to use (a bowl for wastewater is included underneath the tap in this design).

“*My baby throws this water [from the handwashing station] all day long. Because of this problem, I put [the bucket with tap handwashing station] away for a few days. I didn’t add water for a few days.*”

(FGD, Female)

As a potential alternative to overcome these challenges, participants mentioned using their tubewell as a handwashing station by placing soap and a bucket and a mug next to their tubewell:

“*We did not make the bucket [“bucket with tap” handwashing station] but we have a bucket beside the tubewell. We keep water, keep soap [there]. And you are talking about making the bucket [handwashing station]; people’s financial situation is not always the same.*”

(SSI, Male)

Furthermore, during an intervention planning workshop, a CHoBI7 health promoter mentioned observing that some households in the study setting (not enrolled households) prepared their own handwashing station by putting a bottle on the mouth of their tubewell. Placing a bottle on the mouth of a tubewell with the cap off (Figure 2B) slows down the flow-rate of water coming out of the tubewell, allowing the user to wash their hands without having to continuously pump water using their hands. The slow flowrate makes this design child-friendly, easy for children to wash their hands at the tubewell.

Based on these findings, in phase 3, a new handwashing station design, “bottle on the tubewell (mouth)”, was introduced. We recommended that users of the “bottle on the tubewell (mouth)” handwashing station put a soapy water bottle or bar soap next to the tubewell or hanging on this around the next of the tubewell. The “bottle on the tubewell (mouth)” design allowed for a no-cost or low-cost handwashing station—only a plastic bottle is needed for this design, which many households already have in their homes. A demonstration station for this handwashing station design was set up in healthcare facilities during CHoBI7 Scale-up program delivery.

#### 3.1.5. Introduction of a New Handwashing Station Design Using a Plastic Bottle with a Hole

During intervention workshops, research staff mentioned that some households (12%) did not have their own tubewell to prepare a “bottle on the tubewell” handwashing station. To overcome this challenge, one pilot participant mentioned making their own handwashing station with a bottle with a hole at the bottom and hanging a soapy water bottle on a string next to it.

“*In front of the toilet, we could make a hole in a bottle, along with a soapy water bottle, and hang them on a tree with a rope, placing something underneath it [for use as a basin]. That’s all [for the handwashing station].*”

(SSI, Male)

These findings led to the introduction of a third handwashing station design in phase 3, “bottle with a hole at the bottom”, which is a large, 2 L bottle with water and a hole near the bottom and a soapy water bottle hung from a tree or pole (Figure 2C). Like the “bottle on the tubewell (mouth)” design, the hole at the bottom of the bottle allows water to slowly come out for handwashing by loosening the cap, and the design only requires a 2 L plastic bottle. A demonstration station of “bottle with a hole at the bottom” handwashing station was set up at each healthcare facility, and session participants practiced handwashing with soapy water at this location.

#### 3.1.6. WASH Module (Flipbook) Page on Dos and Don’ts of Preparing a Handwashing Station

During intervention planning workshops, the research staff shared photos of handwashing stations prepared by pilot participants. Based on these photos, the research staff identified some challenges with the “bucket with tap” handwashing stations constructed by pilot participants. Some households were installing the tap on their handwashing station too high, making it challenging to use water from the bucket when the volume became low. Other households had their “bucket with tap” handwashing station on the ground, which made it difficult for users to fit their hands under the tap for handwashing. Some participants did not have a lid to cover their “bucket with tap” handwashing station, which at times resulted in children playing with the water inside the bucket (and could pose a drowning risk). Additionally, some participants did not place a bowl underneath their “bucket with tap” handwashing station, resulting in a muddy floor. Some pilot participants also reported that they accidentally made the hole for their handwashing stations too large, causing their tap to not fit properly and leading to leakage:

“*When making [the hole in the bucket], [the rod] is heated in the stove to pierce a hole in it [to fix the tap]. Making the hole is a bit tricky because the rod [iron] is very hot. [The hole] has to be the right size; if it [the rod] is too hot, the hole will end up being too big.*”

(SSI, Female)

Based on these challenges, in phase 5, we added one page in the WASH module (flipbook) on the dos and don’ts of preparing a handwashing station, using photos from pilot households. Instructions were also provided on how to fix broken hardware. This page of the WASH module was well received, with participants reporting that they repaired their broken “bucket with tap” handwashing stations:

“*I saw this [WASH module, flipbook] and I made that basin out of a bucket [“bucket with tap” handwashing station]…I had a broken bucket…I got the glue from the market…and a tap…I attached it [the broken parts of the bucket] with glue, then a cut a hole in one place using scissors [to fix the tap]. That’s how I made it…It was an old bucket, I wouldn’t have used it, [I] would have thrown it away… Later, I thought that instead of throwing it away, I should do this [make the handwashing station].*”

(SSI, Male)

#### 3.1.7. Modified CHoBI7 Scale-Up Program mHealth Component

Through intervention planning workshops, the content of the mHealth component (i.e., mobile messages) was adapted for the rural context of the CHoBI7 Scale-up program prior to the start of the pilot (e.g., “tubewell” instead of “municipal water supply”). All voice and IVR messages were initially sent out at 5 p.m. on Tuesdays and Fridays at the start of the pilot. Included below is an example voice message:

*Assalamulalaikum. This is Dr. Chobi from the hospital. I am calling to remind you to make sure you and your family always wash your hands with soap before eating, feeding your children, and preparing your food, and after using your toilet or cleaning your child’s feces. Use your handwashing station you constructed to help with this. Help young children to wash their hands with soap. Perform these behaviors to keep your family safe from any more severe diarrhea! Share the message with your family!*

 

In the healthcare facility, promoters conducted a training for participants on how to receive mHealth messages and respond to IVR messages, and emphasized the importance of sharing any mHealth messages from Dr. Chobi with all those in the household. Message sharing was also reinforced in mobile messages, as seen in the example above.

#### 3.1.8. Mobile Message Emphasizing Preparing a Handwashing Station During 7-Day High-Risk Period

Some pilot participants mentioned not having the time to prepare their handwashing stations during the 7-day high-risk period. Other participants purchased the tap for their handwashing stations but did not then prepare the handwashing station.

“*I bought the tap and kept it…but I still haven’t made it [fixed the tap to the bucket]… In my opinion, [not making the handwashing station] is just laziness, not because of money, more laziness.*”

(FGD, Female)

Based on these findings, in phase 2, we added automated voice calls the week after in-person intervention delivery, asking households to please prepare their handwashing station as soon as they came home from the healthcare facility, emphasizing the importance of the 7-day high-risk period for diarrheal disease in the household and the health of their children.

#### 3.1.9. Changing Timing of mHealth Message Delivery

Some FGD and SSI participants mentioned that they were busy and not able to receive the phone calls from Dr. Chobi at the originally scheduled time of 5 p.m. Additionally, for pilot participants without their own phones, some reported that other household members were not sharing the mobile messages with them. Pilot participants recommended calls be made in the evening between 7 and 9 p.m., when all household members would be present and could listen to Dr. Chobi’s calls together. To address this challenge, from phase 3 on, we sent out mobile messages at 7:30 p.m.

“*If the phone calls are made after work, I can hear the thing [message] myself and…with the loudspeaker, I can play it for my family or neighbors. If I listen to it by myself, I might not remember everything… And [if I played Dr. Chobi’s call directly to everyone through the loudspeaker], then everyone could hear, everyone could understand.*”

(SSI, Male)

We also received reports from some participants that program text messages were not being received.

“*[We have] two mobiles [phones] but one number is given [to the CHoBI7 program]. But we have never received [text] messages…but the phone [calls] came.*”

(SSI, Female)

To address this challenge, we resent failed text messages using the EngageSPARK mobile platform [19].

#### 3.1.10. Direct Mobile Phone Call by Health Promoters During 7-Day High-Risk Period

During interviews, some participants mentioned that they were not present in the healthcare facility when the CHoBI7 health promoter delivered the intervention and were not clear on or forgot the dosing of chlorine needed to treat water or how to construct a handwashing station for their household.

“*No, I was not [at the healthcare facility]. If you explain [how to make a handwashing station] again now, I will make it.*”

(SSI, Male)

Based on this finding, in phase 5, we introduced two direct calls to households from health promoters on Day 2 and Day 6 of the high-risk period, providing additional guidance on how to prepare a handwashing station and treat water with chlorine tablets. The CHoBI7 testimonial video that was shown in the healthcare facility and photos of self-made handwashing station designs were also shared by health promoters with households using WhatsApp following these direct calls. This modification was well received and helped provide guidance to those that were not present during the CHoBI7 healthcare facility visit.

“*When I heard [about making the handwashing station], the next day I made it [“bucket with tap” handwashing station]. From where I got the training, I was sent pictures and videos via WhatsApp.*”

(SSI, Male)

#### 3.1.11. Cue Card and Mobile Messages on Chlorine Tablet Dosing Instructions

Several pilot participants reported that they were confused on how to treat their stored household drinking water.

“*Initially, I was confused about whether to add one or two [chlorine] tablets, [and] how many liters of water.*”

(SSI, Female)

Our study site had high iron levels in groundwater (>5 mg/L Fe). Iron has a high chlorine demand, reducing the free chlorine available to disinfect stored drinking water [24]. During the initial two phases of the pilot, we observed that <10% of pilot households had free chlorine >0.2 mg/L (WHO cutoff) during the 7-day period after enrollment [25]. Based on these findings, we developed a cue card with chlorine tablet dosing instructions based on the size of different water storage vessels. The chlorine dosing instructions were based on a series of chlorine dosing experiments in households with a range of iron concentrations in our pilot area (unpublished). Iron concentrations were measured using the Hach Iron color disc test kit (model IR-18B). Experiments were conducted where iron concentrations were measured, and chlorine was added at increasing doses (bucket chlorination test). From the third phase of the pilot, a cue card was provided on the updated recommended chlorine dosing instructions based on the type of water storage container used (Supplementary Figure S3). After making changes to the dosing of chlorine tablets in phase 2 of the pilot, the proportion of households with free chlorine during the 7-day period after enrollment increased to 55% in phase 3. The cue cards with chlorine dosing instructions were well received by pilot participants.

“*[My husband] set the pictures [cue cards and stickers] in such a way that as soon as I put the water jug in that place, I see that I need to add the tablet. That’s how I remember.*”

(SSI, Female)

#### 3.1.12. Mobile Messages Emphasizing That Iron Filters Do Not Remove Germs

Pilot participants reported making self-made iron filters (using sand and brick pieces/stones in a clay pot) (Supplementary Figure S4) to remove the iron from drinking water, as it impacts the taste and appearance of water. Some participants reported that self-made iron filters also removed microbial contamination.

“*Before, I had a clay/mud-made filter [to filter out iron]. I made this filter with sand, pieces of bricks, and cloth at the bottom in the pot, then making holes. By filtering this way, water becomes germ-free. The water becomes clean.*”

(SSI, Female)

Based on this finding, we explained in mobile messages from phase 3 onwards that iron filters do not remove the germs that cause diarrhea and recommended that households use their self-made iron filters first to remove the iron in their water and then use a chlorine tablet to remove germs from their water to make it safe for drinking. This was well received in the subsequent phase, with participants reporting that using an iron filter does not make the water germ-free.

“*[This] filter is made by hand with sand and clay. [With this filter], then there is less iron [in the water]. It is not safe… It is not germ-free; germs are definitely there. To make it germ-free one must add [chlorine] tablets to the water.*”

(SSI, Female)

Some recipients chlorinated their drinking water prior to using their self-made iron filter, which reduced free chlorine concentrations and presented a challenge with ensuring microbially-safe drinking water. Instructions were provided during the in-person session in the healthcare facility to use self-made iron filters prior to adding chlorine tablets.

#### 3.1.13. Mobile Messages Emphasizing That Chlorine Tablets Should Be Used Beyond the 7-Day High-Risk Period

Several pilot participants mentioned during semi-structured interviews that chlorine tablets were no longer needed after the 7-day high-risk period for diarrhea was over, with some mentioning that they were saving chlorine tablets for future diarrhea episodes.

“*The rest of the tablets [chlorine] have been kept, my family [wife] can confirm this …Everyone in the family has recovered from that [diarrheal disease], so, they were no longer needed. Based on that, she [his wife] kept some, I think, few of the tablets have been kept.*”

(SSI, Male)

Other pilot participants referred to the chlorine tablets as medicine, stating that since they have already used the tablets, they are cured.

“*[They] gave us medicine [chlorine tablets]. We took the medicine by adding [the tablets] to water in the way we were told [as part of the intervention]. We benefitted from this but could not finish all [of the tablets]. [The tablets] worked well after taking some of them… we were cured by taking some of them. Since we recovered, we don’t need to take them anymore.*”

(SSI, Male)

Based on these findings, we emphasized in mobile messages starting in phase 2 that it was important to use all chlorine tablets provided, not only during the 7-day high-risk period. Messages also emphasized that consuming chlorine tablets now does not prevent future diarrhea if you stop using the chlorine tablets. We explained that chlorine tablets are not medicine.

#### 3.1.14. WASH Module (Flipbook) Clarifying That Arsenic- and Iron-Free Water Can Still Have Germs That Cause Diarrhea

Several participants mentioned that their water was safe to drink because it had no arsenic or iron and that they sometimes drank water without using chlorine tablets.

“*We drink [water] with [chlorine] tablets, even so [without adding the tablets] we drink it directly from it [tubewell]. Our tubewell water has no arsenic, even no iron, [it is] very fresh.*”

(SSI, Male)

Based on this finding, we emphasized in the WASH module (flipbook) from phase 3 onward that even tubewell water with no arsenic or iron can have germs that could cause diarrhea.

### 3.2. Implementation Outcomes

**Figure 3:**
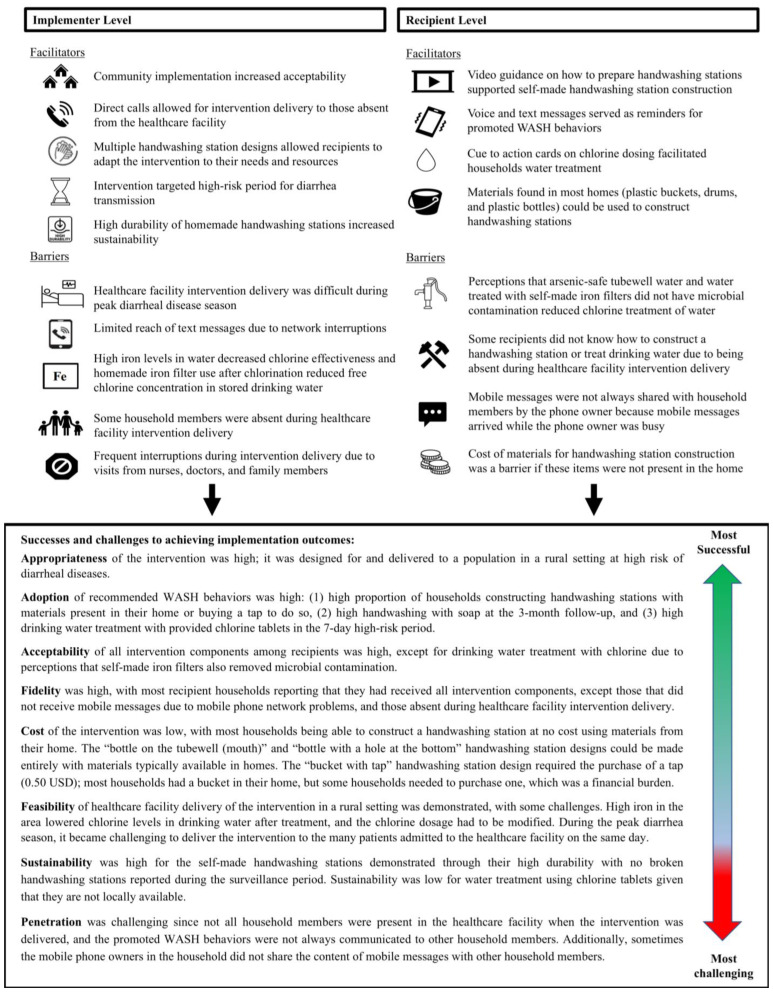
Graphical summary of study results.

The implementation outcomes from SSIs and FGDs are summarized in Table 2. A graphical summary of the facilitators and barriers to study implementation along with an overview of the most successful and challenging aspects of study implementation is shown in Figure 3.

#### 3.2.1. Recipient-Level Implementation Outcomes

Acceptability of all components of the CHoBI7 Scale-up program delivery among recipients was high. However, the belief that because tubewells were arsenic- and iron-free, they were also free from germs reduced the acceptability of water treatment among some participants. Adoption of the targeted handwashing with soap and water treatment behaviors was high during the 3-month pilot period, as evidenced by the intervention uptake reports. The appropriateness of the CHoBI7 Scale-up program was high, with recipients reporting that diarrhea was a problem for their households and this program was important to reduce further diarrhea. However, there appeared to be more concern about iron contamination than germs in tubewell water. The cost of constructing a handwashing station was not a major barrier for most recipients, with most constructing handwashing stations using materials already in their home. The fidelity of intervention delivery was high among recipients, both for those receiving the intervention through the WASH module (flipbook) if they were present in the healthcare facility and for those receiving direct voice calls if they had been absent from the healthcare facility. However, sometimes, text messages were not received. Construction of handwashing stations by recipients was found to be a feasible approach. Penetration was challenging since some household members were not present in the healthcare facility when the intervention was delivered, and some were unavailable when calls came to their mobile phone. Additionally, intervention content sent by mobile messages was not always shared with those without their own mobile phones. The program was found to be sustainable, with recipient handwashing with soap and water treatment practices sustained to the 3-month follow-up, as evidenced by the intervention uptake reports.

#### 3.2.2. Implementer Level Implementation Outcomes

Implementers of the CHoBI7 Scale-up program reported high acceptability among recipients. The use of health promoters residing in the community was viewed as an important aspect of intervention acceptability and high uptake of the intervention. Adoption was viewed by implementers to be high, as evidenced by quantitative intervention uptake reports of spot checks and 5 h structured observations. However, drinking water treatment using chlorine tablets was a challenge because of high iron levels in water, which reduced the free chlorine present in drinking water for disinfection, and using self-made iron filters after chlorination reduced the free chlorine concentration in stored drinking water further. Recommending higher dosages of chlorine overcame this challenge; however, there was still the perception by recipients that an iron filter removed germs, so there was no need for further water treatment.

“*Their handwashing habit is satisfactory, but they don’t have any habit to boil their drinking water… As they have a lot of iron in the village area of Manikganj, they use a local filter [to remove iron]…and their idea is that water is safe as long as it is arsenic-free, and the local iron filter is used to remove the iron.*”

(Female Health Promoter)

The iterative approach for piloting was viewed by implementers as important because it allowed challenges that occurred with intervention implementation to be addressed in the subsequent pilot phase to ensure the appropriateness of program delivery. Implementers reported that the intervention had a low cost, with most diarrhea patients’ households stating that they already had the materials needed to construct a handwashing station already in their home. The main cost was buying a tap, which was 50 taka (USD 0.50). Taps were available at most local markets and were not mentioned as a financial burden by households.

Implementers emphasized the importance of having multiple designs of handwashing stations, which allowed recipients to adapt their handwashing station design to their own needs and the availability of materials in their home. Implementers mentioned that the “bottle on the tubewell (mouth)” and “bottle with a hole at the bottom” handwashing station designs could be made completely with materials available in the home of recipients and did not have an associated cost. This was viewed as valuable given that some households did not have a bucket to make the “bucket with tap” handwashing station design. Overall, implementers reported that intervention fidelity was high, with most recipient households reporting receiving all intervention components. However, implementers faced challenges delivering the CHoBI7 Scale-up program in healthcare facilities due to frequent interruptions from family members, healthcare facility staff, and incoming calls to the patient’s mobile phone.

“*The doctor is visiting, we have to stop then and take a break. The nurse is coming, we have to take a break here, too. Or the patient’s household members are coming and have to take a break here.*”

(Female Health Promoter)

Additional challenges were reported by implementers. One was that while implementers reported that the intervention was feasible, during the peak diarrhea season, it became very challenging to deliver the intervention to the many patients being admitted to the healthcare facility on the same day. A further challenge was that diarrhea patients often had difficulty focusing on intervention delivery in the health facility due to their illness. Implementers also mentioned penetration to be a challenge for program delivery because many household members of diarrhea patients were not present in the healthcare facility at the time of intervention delivery.

“*The highest number of participants we are getting from each family is up to 1 or 2 members [for the healthcare facility based intervention]. If we are very lucky, we get three people but we don’t get more than that. …Especially the head of the family, we don’t get him [during the healthcare facility intervention delivery].*”

(Female Health Promoter)

Additionally, implementers mentioned that recipients sometimes did not receive automated voice, IVR, and text messages due to disruptions in the mobile network. Direct calls to diarrhea patient households during the 7-day high-risk period were viewed as an effective approach to overcome this challenge.

Implementers had four key recommendations for program scalability. First, they stated the need for multiple options for handwashing station designs for households since one size will not fit all. Second, the iron levels in households need to be carefully considered when making recommendations on chlorine dosing. The recommended dosing will likely vary by geographic location. Third, implementers recommended ensuring the availability of chlorine tablets in the local market, as boiling water is not a common practice among rural households.

“*When they [recipients] first received the chlorine [tablets], they thought that they could buy it from the shop or pharmacy. But it is not available in the market. We have to ensure this [chlorine tablet availability]—that if they want to buy it, they can. If we are able to ensure this [chlorine tablet availability], then we can sustain it. Otherwise, they will drink the tubewell water directly. They will not boil their water. This is something we should pay attention to.*”

(Female Health Promoter)

Finally, implementers recommended further engagement of doctors and nurses in the healthcare facilities where the CHoBI7 Scale-up program is being delivered by having them mention one or two sentences about the importance of CHoBI7 delivery during their patient rounds. This could help increase acceptability of the intervention among recipients.

“*I think we have to work together along with the doctors and nurses. If we work together it would be more effective…We have to provide training to doctors and nurses, and we also need to work together; then, it would be good. When a doctor or nurse visits a patient during rounds, then they can also discuss this matter [the CHoBI7 program]; this would be beneficial.*”

(Female Implementer)

### 3.3. Quantitative Pilot Study Findings

In the pilot study, 275 participants were recruited from 64 diarrhea patient households (49 CHoBI7-arm (intervention-arm) households and 15 standard-arm households). Among the 49 households, 50% of CHoBI7-arm households in pilot phase 1 constructed their own handwashing station, 27% in phase 2, 82% in phase 3, 80% in phase 4, and 90% in phase 5. Twenty-seven households constructed “bucket with tap” handwashing stations, one household constructed a handwashing station with a 5 L oil container with tap (new model built by household), five households constructed a “bottle with a hole at the bottom” handwashing station, and one constructed a “bottle on the tubewell (mouth)” design (Supplementary Figure S2). Four households constructed two handwashing stations. There were no handwashing stations constructed in the 15 standard-arm households.

In intervention households, handwashing stations with water and any soap present increased at the Day 7 follow-up from phase 1 to 5 from 13% to 50%, at the Month 1 follow-up from 25% to 50%, and at the Month 3 follow-up from 0% at 50% (Supplementary Table S3). For handwashing with soap during the 5 h structured observation, the Day 7 follow-up was similarly high at phase 1 and 5 with 53% and 50% handwashing with soap, respectively (compared to 23% in the standard arm). At the Month 1 follow-up, handwashing with soap increased from 24% in phase 1 to 36% in phase 5 (compared to 6% in the standard arm), and at the Month 3 follow-up, this decreased from 50% in phase 1 to 32% in phase 5 (compared to 22% in the standard arm). For free chlorine >0.2 mg/L in stored drinking water, in intervention households at Day 7, this increased from 0% to 56% from phase 1 to 5; at the Month 1 follow-up, this increased from 0% to 38%; and at the Month 3 follow-up, this increased from 0% to 63%. In addition, no intervention households reported that their handwashing station broke during the 3-month pilot period. This finding demonstrates the high durability of the handwashing stations constructed by diarrhea patient households.

The process evaluation of the mHealth program found in phase 5 that 73% of voice messages and 79% of IVR messages were fully listened to, and 88% of text messages were received. This result shows the high fidelity of the mHealth program despite the phone network connectivity challenges identified in the qualitative findings.

## 4. Discussion

This study reports on community-centered formative research using an implementation science framework to adapt the CHoBI7 mHealth program to a rural context with a modified, lower-cost, more scalable diarrhea prevention package that only provided chlorine tablets and soapy water (CHoBI7 Scale-up program). Interviews and FGDs with diarrhea patients and their household members (intervention recipients) and program implementers informed our understanding of the facilitators and barriers to the promoted WASH behaviors as well as the implementation of the CHoBI7 Scale-up program. The iterative design of the pilot study allowed for the identified barriers to be addressed in the subsequent pilot phases. Furthermore, applying an implementation science framework supported identification of program successes and challenges [22]. During the final phase of the pilot study, the majority of intervention households (90%) constructed their own handwashing station and had free chlorine >0.2 mg/L (63%) at the 3-month follow-up. These findings demonstrate that the CHoBI7 Scale-up program with a single in-person health promoter visit in the healthcare facility, a diarrhea package with chlorine tablets and soapy water, and an mHealth program with weekly voice and text messages was effective in increasing WASH behaviors among a population at high risk for diarrheal diseases in a rural setting in Bangladesh.

The key successes of the CHoBI7 Scale-up program were the high acceptability of the intervention among recipients, high adoption of the promoted WASH behaviors, and high appropriateness of the intervention for the rural context and for a high-risk population for diarrheal diseases. These successes were facilitated by the multiple, self-made handwashing station designs that were promoted, which allowed recipients to select the design that best suited their needs. In addition, successes were facilitated by videos that provided guidance on how to construct handwashing stations, voice calls and text messages that allowed for intervention content to be shared with those not present in the healthcare facility and serve as reminders of the promoted WASH behaviors, and the cue-to-action card provided on the context-specific chlorine dosing needed for water treatment. Finally, engaging health promoters who resided in the intervention communities allowed for higher intervention acceptability. These findings highlight the importance of conducting formative research to purposefully adapt WASH interventions for target populations.

There were several challenges to intervention implementation identified during our formative research. First, for intervention fidelity, the high patient volume in district and sub-district healthcare facilities during peak diarrhea seasons made it challenging for health promoters to deliver the intervention content to all diarrhea patients. Additionally, promoters faced challenges delivering the CHoBI7 Scale-up program in healthcare facilities due to frequent interruptions from family members, healthcare facility staff, and incoming phone calls to the patient. Further engagement of healthcare providers in program implementation by having them mention the importance of the CHoBI7 program to prevent diarrhea during their patient visits could help to minimize the impact of interruptions from these visits. Therefore, to ensure a scalable approach for the delivery of CHoBI7, government healthcare facility staff should be engaged in program delivery. A further challenge to intervention fidelity was that while mobile messages were considered beneficial to share intervention content for those who were not present in the healthcare facility, network issues sometimes hampered the delivery of text messages. For challenges related to penetration, not all household members were present during intervention delivery in the healthcare facility. Moreover, phone owners in recipient households did not always share mobile messages with other household members. Mobile message sharing needs to be further reinforced and supported during program implementation. Finally, a major challenge to program sustainability was the lack of locally availability chlorine tablets in our study setting. This is particularly important given the low uptake of boiling water in Bangladesh at our site due to disliking the taste of boiled water and the time needed for boiling (unpublished data). All of these challenges will be important to consider during intervention implementation if the CHoBI7 program is taken to scale.

Nearly all recipients (90%) of the CHoBI7 Scale-up program in the final phase of piloting constructed their own handwashing stations. During pilot phases, the majority of recipients (55%) constructed a “bucket with tap” handwashing station design, which required the purchase of a tap for USD 0.50 from the local market. Households that did not have an extra bucket in their home to prepare the “bucket with tap” design reported making either the “bottle on the tubewell (mouth)” or “bottle with a hole at the bottom” designs, which could be made solely with materials from their home with no construction cost. Promoting multiple handwashing station designs allowed for households to adapt their construction based on the materials present in their home and household members’ needs. In addition, high durability of the handwashing stations constructed by recipients was observed, with no broken handwashing stations reported during the surveillance period. These findings are consistent with a previous study in rural Bangladesh that promoted construction of self-made handwashing stations and found high uptake [26]. However, this study included frequent home visits by a health promoter for intervention delivery. Our findings demonstrate the feasibility of a modified diarrhea prevention package, which promotes construction of a self-made handwashing station instead of relying on the provision of a premade handwashing station. During our previous interviews with government stakeholders in the Ministry of Health and Family Welfare, distribution of handwashing stations in healthcare facilities to diarrhea patients was not considered an approach that the Bangladesh government could scale [13]. Therefore, this study builds important evidence on a scalable approach to deliver the CHoBI7 program in Bangladesh.

Over half of recipient households in the final pilot phase had stored-water chlorine levels at the recommend level (>0.2 mg/L). This finding is despite the elevated iron concentrations in drinking water, which posed a significant barrier to program implementation. Challenges with high iron concentrations in water reducing the effectiveness of chlorination interventions has been noted in previous research from Bangladesh [24]. Some recipients in our study already used self-made iron filters and incorrectly thought that the iron filters also removed microbial contamination. This emerged as an important challenge for acceptability of water treatment with chlorine tablets. Additionally, some recipients chlorinated their drinking water prior to using their self-made iron filter, which reduced water chlorine concentrations (chlorine was absorbed inside the iron filter and thereby removed from the water) and presented a challenge to intervention fidelity. Through the iterative pilot study, we were able to address these challenges by identifying chlorine dosing that was appropriate for households with elevated iron. We also adapted WASH module (flipbook) pages and mobile messages to explain that iron filters do not remove germs and that if an iron filter is used, it should be used prior to chlorination. These adaptations to the water treatment component of the intervention resulted in the high proportion of households with chlorine >0.2 mg/L in stored water in the final pilot phase. Without formative research to adapt the CHoBI7 mHealth program to a rural context, the impact of iron on chlorination would likely have been overlooked.

The formative research conducted in this study builds on our previous CHoBI7 research in urban Bangladesh, which found that this program was effective in increasing WASH behaviors and reducing diarrhea and cholera [12,18,27]. The findings from the current study demonstrate that a targeted WASH intervention for a high-risk population for diarrheal diseases can be effective in facilitating WASH behavior change in a rural setting in Bangladesh. Consistent with our findings, a prospective cohort study in the Democratic Republic of the Congo (DRC) found delivery of hygiene kits (chlorine tablets, bar soap, and a handwashing station) to suspected cholera-case households significantly reduced fecal contamination in stored household drinking water compared to households not receiving these kits [28]. Additionally in the DRC, our recent RCT of the Preventative-Intervention-for-Cholera-for-7-days (PICHA7) WASH program delivered to diarrhea patients in healthcare facilities with a similar WASH and mHealth package resulted in significant increases in water treatment and handwashing with soap behaviors [29]. Research is needed on the effectiveness of the CHoBI7 Scale-up program in other settings globally. There is also limited research on the policy-enabling environment needed for successful scale-up of this type of WASH program, which is also crucial for successful program implementation. Finally, additional research is needed on how mHealth programs can be best integrated into health systems to increase health protective behaviors using approaches similar to CHoBI7.

This study had several key strengths. First, the use of both quantitative methods (unannounced spot checks and structured observations) and qualitative methods (SSIs, FGDs, and workshops) to conduct formative research which engaged communities to adapt the CHoBI7 mHealth program to a rural context. This approach allowed us to gain a robust understanding of the facilitators of and barriers to program implementation. Second, applying an implementation science framework helped us to identify the key successes and challenges of program implementation. Third, the iterative process used for adapting the CHoBI7 mHealth program to the rural context through multiple phases of piloting allowed for the barriers to program implementation to be addressed and tested. Finally, implementing the modified diarrhea prevention package during piloting allowed us to determine the feasibility of recipients constructing their own self-made handwashing facilities.

This study also had some limitations. First, the pilot of the CHoBI7 Scale-up program followed households for 3 months, not allowing us to assess the long-term impact of the program on WASH behaviors. This should be investigated in future studies, the current study focused on the period of highest risk for diarrheal diseases in these households. Second, the pilot included only one district in Bangladesh, limiting the generalizability of the study findings. Research is needed in diverse geographic areas and with varying socio-economic gradients to assess the feasibility for adaptation to scale-up throughout the country. 

## 5. Conclusions

This community-centered formative research using an implementation science framework allowed for the adaptation of the CHoBI7 mHealth program to a rural context, with a modified, lower-cost, scalable diarrhea prevention package. High appropriateness, acceptability, and adoption of this program was observed, with most recipients constructing their own self-made handwashing stations using materials in their home. Increased water treatment and handwashing with soap behaviors were also observed. Engaging healthcare providers in program implementation, providing multiple options for self-made handwashing stations, ensuring the availability of chlorine tablets in the local market, and considering iron levels when making recommendations on chlorine dosing were identified as important considerations for program scaling. These findings provide valuable documentation of the process of adapting the CHoBI7 mHealth program to a rural context and demonstrate the important role of formative research for tailoring WASH programs to new contexts. We are currently partnering with the Bangladesh Ministry of Health and Family Welfare to scale the CHoBI7 mHealth program in rural and urban areas of Bangladesh, based on the findings from this study and our previous formative research and RCTs [7,12,13,18,20,26,27,30]. An ongoing RCT of this CHoBI7 mHealth program is investigating the health impact of intervention delivery .

## Figures and Tables

**Table 1:** CHoBI7 pilot study baseline demographics by study arm.

Baseline Demographics	Intervention Arm			Standard Message Arm		
	%	*n*	N	%	*n*	N
Participants enrolled		219			56	
Children < 5 years of age	28%	62	219	29%	16	56
Child caregivers	44%	97	219	38%	21	56
Female	57%	125	219	63%	35	56
Households		49			15	
Individuals living in household	5 ± 2 (3–10)			5 ± 1 (3–6)		
(Mean ± SD (Min–Max))						
Household member literacy	92%	45	49	93%	14	15
Child caregiver literacy	94%	46	49	93%	14	15
Unimproved latrine	37%	18	49	20%	3	15
Household refrigerator ownership	65%	32	49	60%	9	15
Electricity	100%	49	49	100%	15	15
Presence of Soap in:						
Kitchen area	8%	4	49	13%	2	15
Toilet area	8%	4	49	27%	4	15
No household income source	2%	1	49	0%	0	15
Water source type:						
Ground water	94%	46	49	93%	14	15
Pond	0%	0	49	0%	0	15
Dug well	4%	2	49	0%	0	15
Piped water supply	2%	1	49	7%	1	15
Collected rainwater	0%	0	49	0%	0	15
River	0%	0	49	0%	0	15
Canal	0%	0	49	0%	0	15
Household roof type:						
Concrete	4%	2	47	7%	1	15
Tin	96%	45	47	93%	14	15
Hay	0%	0	47	0%	0	15
Leaves	0%	0	47	0%	0	15
Others	0%	0	47	0%	0	15
Sleeping rooms in household:						
1 sleeping room	20%	10	49	27%	4	15
2 sleeping room	41%	20	49	40%	6	15
3 sleeping room	24%	12	49	20%	3	15
4 sleeping room	14%	7	49	13%	2	15
Numbers of sleeping rooms	2.44 ± 0.99 (1–4)			2.19 ± 0.98 (1–4)		
(Mean ± SD (Min–Max))						

Household member literacy defined as at least one household member who could read and write. SD: standard deviation.

**Table 2:** CHoBI7 Scale-up Program Implementation Outcomes.

Implementation Outcome	Implementer Perceptions	Recipient Perceptions
**Acceptability**	Implementers reported that recipients generally accepted CHoBI7 delivery and viewed it as important to reduce the spread of diarrheal diseases. Development of three separate handwashing station models based on participant feedback increased acceptability of the intervention. The use of health promoters residing in the community was also viewed as an important aspect of intervention acceptability.	Most recipients appreciated CHoBI7 delivery at healthcare facilities to help prevent diarrhea in their households. Most recipients reported liking the handwashing stations they constructed as part of the intervention. Some reported that their tubewells were arsenic- and iron-free, and therefore, drinking water did not need to be treated with chlorine tablets. Most recipients liked the voice calls sent to their mobile phone from the mobile health program. Cue cards were considered valuable to promote WASH behaviors.
**Adoption**	Implementers stated that many households reported preparing their own handwashing stations during direct calls. High iron levels in water decreased chlorine effectiveness, and using homemade iron filters after chlorination reduced free chlorine concentration in stored drinking water.	Reported adoption of handwashing with soap and water treatment behaviors was high during the 7-day high-risk period and sustained to the 3-month follow-up (corresponding with structured observation and unannounced spot check reports). A high proportion of households reported preparing their own handwashing stations.
**Appropriateness**	Implementers viewed the adapted CHoBI7 program as relevant for the rural context. The iterative pilot study was viewed as useful to tailor intervention development to this context. Intervention delivery timing to severe diarrhea patients and their accompanying household members was viewed as appropriate.	Recipients generally felt that diarrheal diseases were a problem for their household. Handwashing with soap and water treatment were viewed as important interventions to protect the health of their families. There appeared to be more concern around iron contamination (aesthetic qualities of the water) than germs in tubewell water.
**Cost**	Implementers reported that the intervention had a low cost, with most households reporting already having intervention materials in their home. The cost of materials like buckets was a barrier to constructing the “bucket with tap” handwashing station for some recipients that did not have these items in their home. However this was overcome by recommending 3 different models of self-made handwashing stations, two of which did not require a bucket.	Overall cost was not considered a major barrier to constructing a handwashing station. The cost of buckets to construct the “bucket with tap” handwashing stations was a concern for some recipients who did not have one already in their home. However, most recipients constructed stations at no additional cost using materials from their homes.
**Fidelity**	Implementers faced challenges delivering CHoBI7 in healthcare facilities due to frequent interruptions from healthcare facility staff and visiting family members. Implementers also faced challenges with sending voice and SMS messages due to network problems. Most households achieved high stored-water chlorine levels. However, elevated iron concentrations in drinking water and using self-made iron filters initially presented a major challenge for intervention fidelity for water treatment.	Recipients present in the healthcare facility during intervention delivery reported receiving healthcare facility delivery of the CHoBI7 WASH module (flipbook) by a health promoter. Most recipients reported receiving voice calls; however, some recipients reported not receiving text messages.
**Feasibility**	Implementers reported that both healthcare facility visits and sending voice and SMS messages for intervention delivery was feasible. However, during the peak diarrhea season it became very challenging to deliver the intervention in the healthcare facilities due to many patients being admitted to the on the same day.	Healthcare facility delivery of CHoBI7 in a rural setting was shown to be feasible. Recipients constructing their own handwashing stations was found to be a feasible approach as well as delivery of CHoBI7 voice and SMS messages in a rural setting.
**Penetration**	Implementers reported that reaching all household members for intervention delivery in the healthcare facility was a challenge. Direct phone calls were valuable for reaching household members of diarrhea patients who were not present in the healthcare facility.	Some household members were not present in the healthcare facility when the intervention was delivered, and some were unavailable when calls came to their mobile phone. Some household members were not able to share mobile messages with their household because they were busy outside of the home. The content of the WASH module (flipbook) was not always shared with those not present in the healthcare facility when the intervention was delivered.
**Sustainability**	Implementers mentioned chlorine tablets were not locally available.	Recipient handwashing with soap and water treatment practices were sustained to the 3-month follow-up from both participant reports and from unannounced spot checks and structured observations.
**Scalability**	Implementers emphasized the need for multiple options for handwashing station models for households. For chlorination, implementers reported that iron levels in household water need to be carefully considered when making recommendations on chlorine dosing, and chlorine tablets need to be available in the local market for scaling. It was recommended that there be further engagement of healthcare facility staff in CHoBI7 program delivery.	*This was not explored at the recipient level*.

## Data Availability

The anonymized data presented in this study are available upon request from the corresponding author.

## Supplementary Materials

The following supporting information can be downloaded at https://www.mdpi.com/article/10.3390/ijerph22020170/s1: Supplementary Figure S1: Summary of Formative Research Activities; Supplementary Figure S2: Handwashing Stations Prepared by Households; Supplementary Figure S3: Instructions on chlorine tablet dosing; Supplementary Figure S4: Outside Appearance of Self-made Iron Filters; Supplementary Table S1: Semi-structured interviews and focus group discussions with study participants and implementers; Supplementary Table S2: Overview of CHoBI7 Scale-up intervention modifications over 5 pilot phases; Supplementary Table S3: CHoBI7 quantitative study findings.
