# Supplementary material for: Formative Research for Adapting the Cholera-Hospital-Based-Intervention-for-7-Days (CHoBI7) Water Treatment and Hygiene Mobile Health Program for Scalable Delivery in Rural Bangladesh
Source: Int J Environ Res Public Health. 2025 Jan 26;22(2):170. doi: 10.3390/ijerph22020170 (PMC11855457; doi:10.3390/ijerph22020170)
Supplement: Supplementary file 1 [file ijerph-22-00170-s001.zip › ijerph-3088921-supplementary.pdf]

CHoBI7 Scale-Up Manuscript Supplementary Figures

Supplemental Figure S1: Summary of Formative

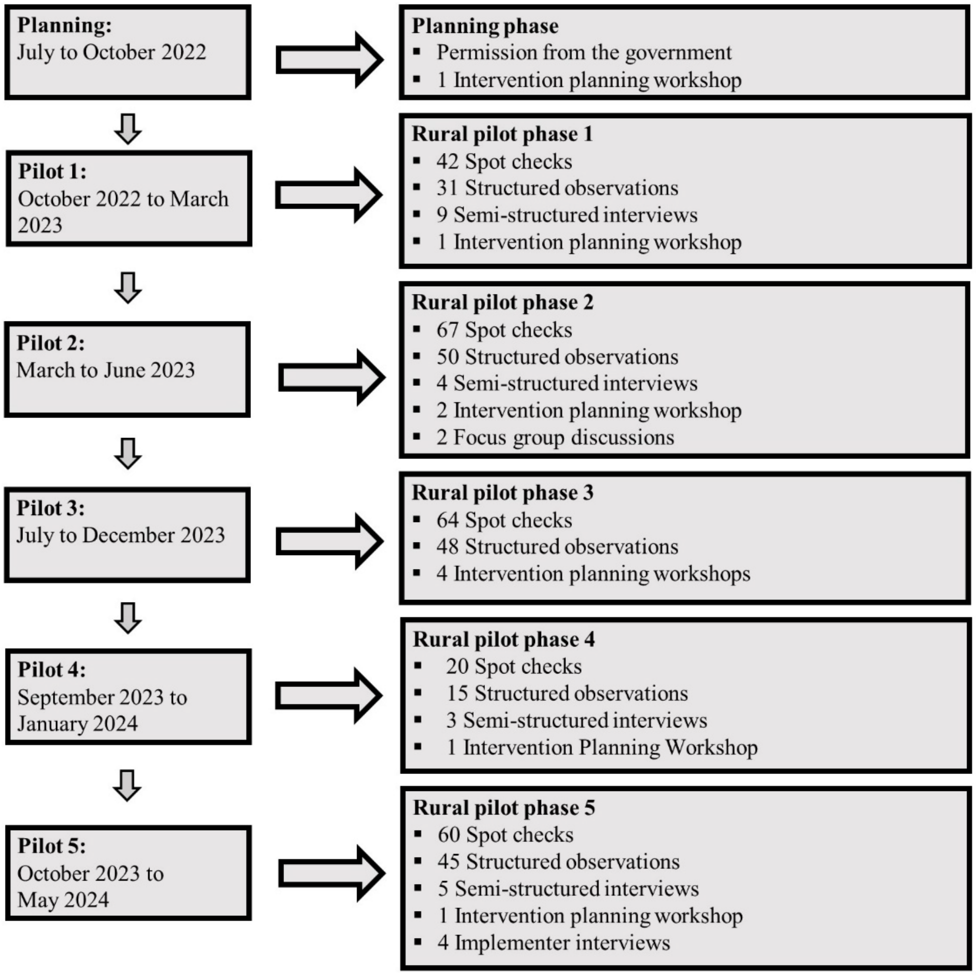

Supplementary Figure S2. Handwashing Stations Prepared by Pilot Households

Bucket with Tap Handwashing Station Design

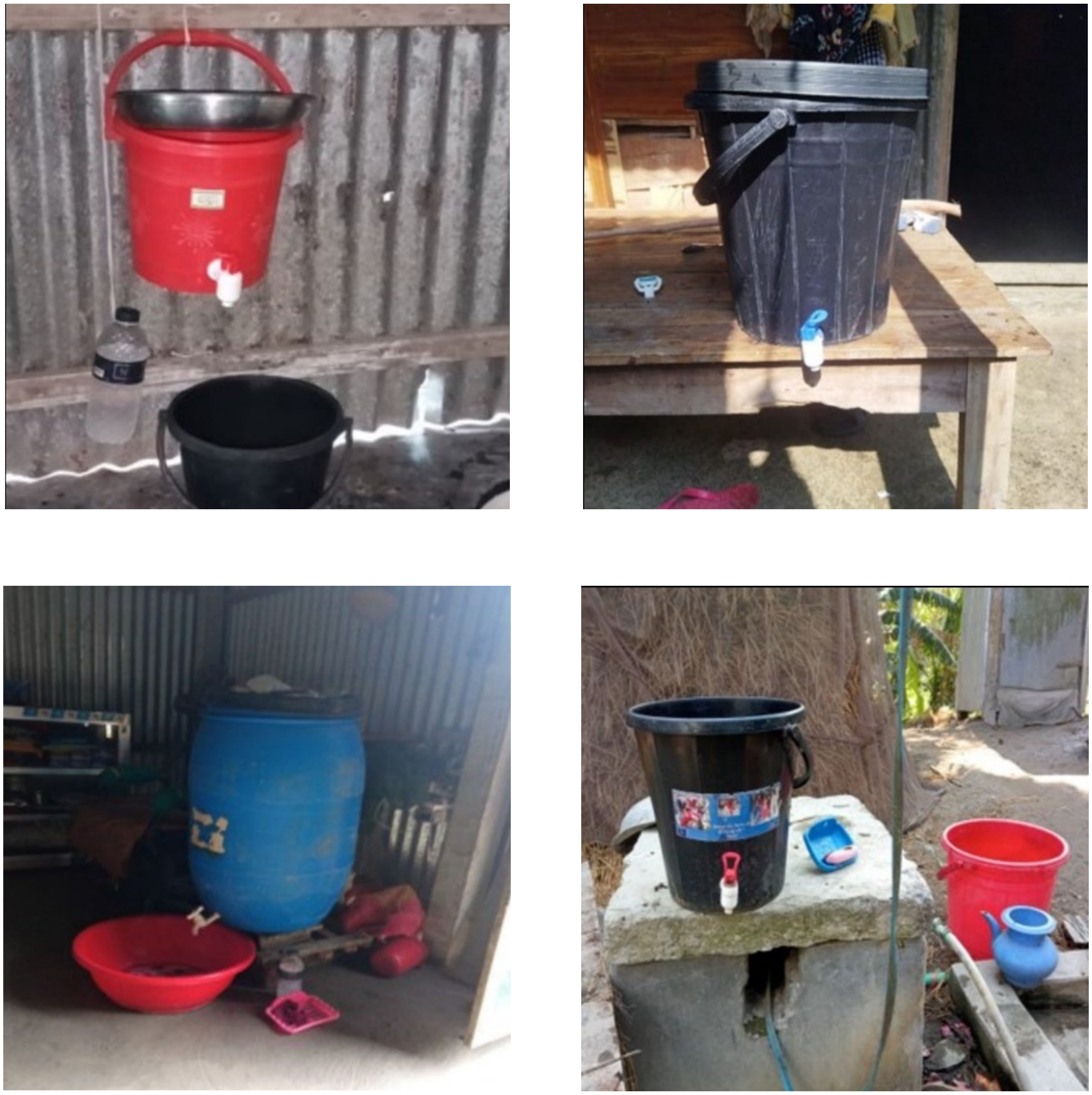

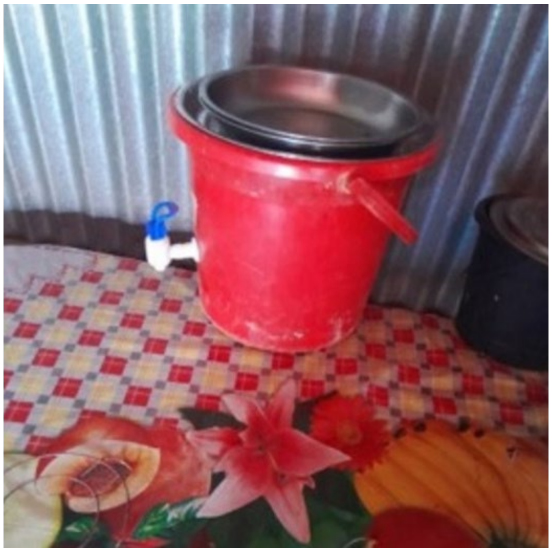

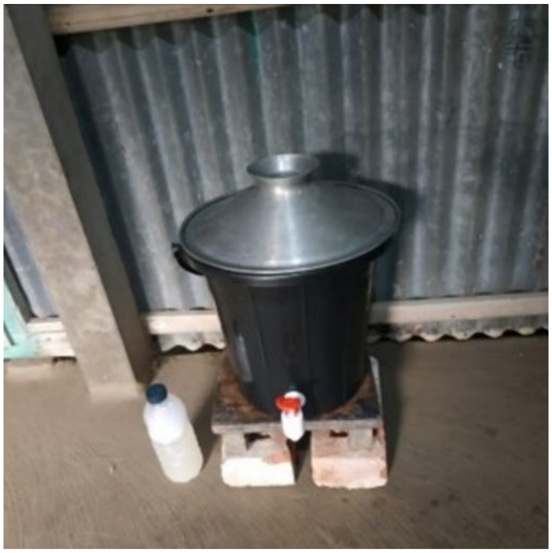

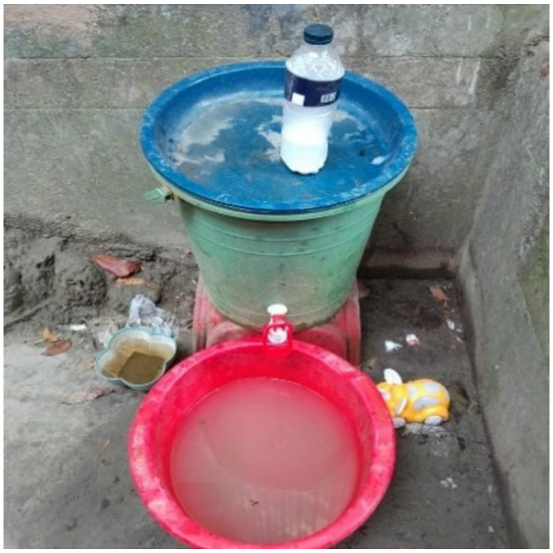

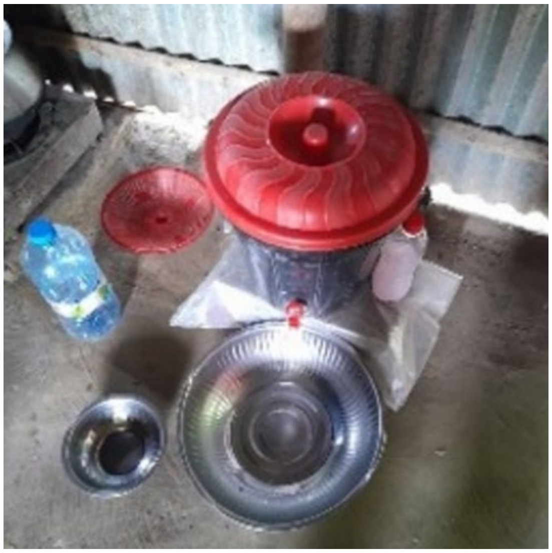

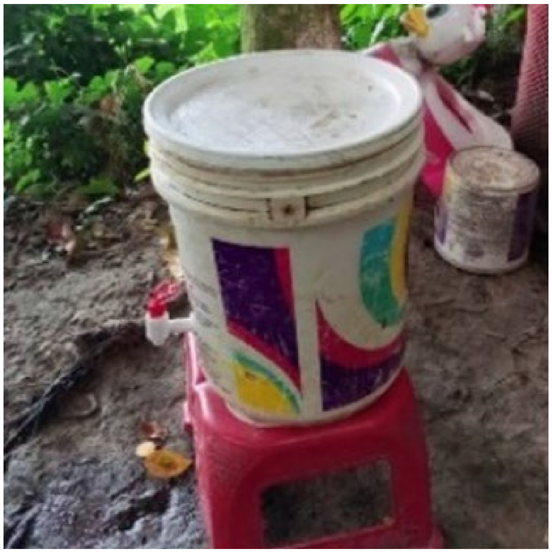
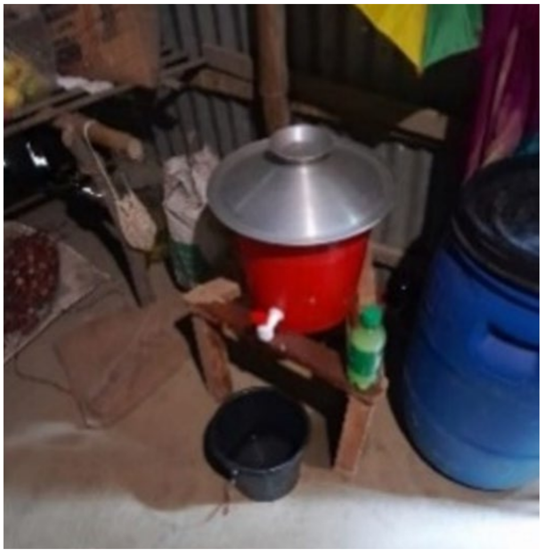
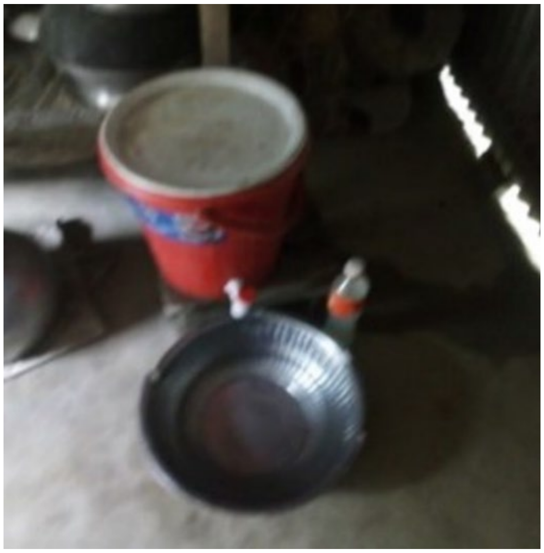
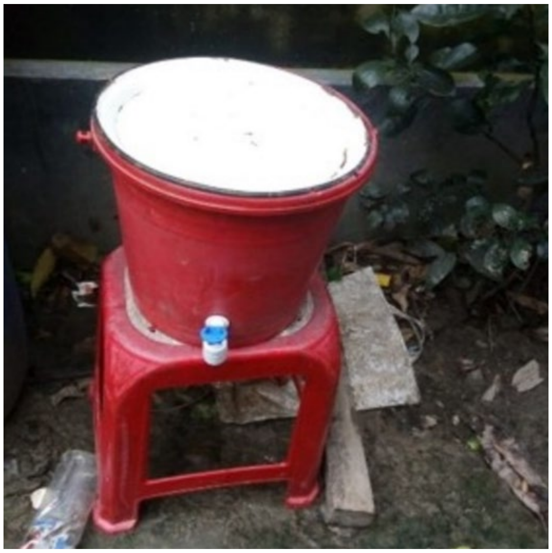

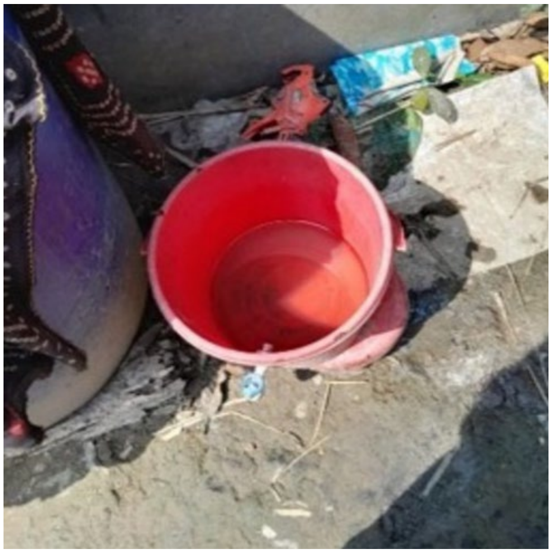
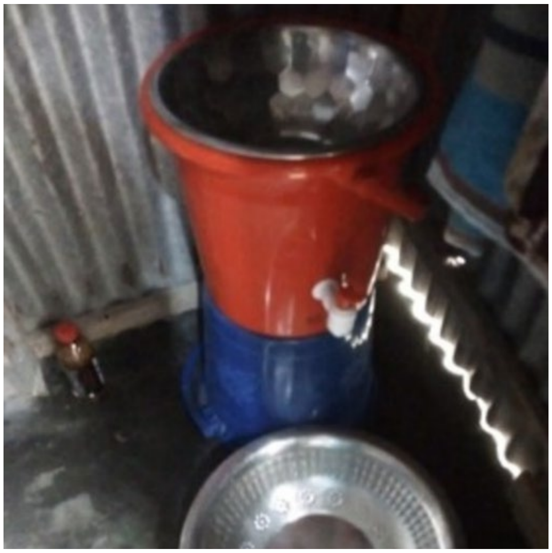
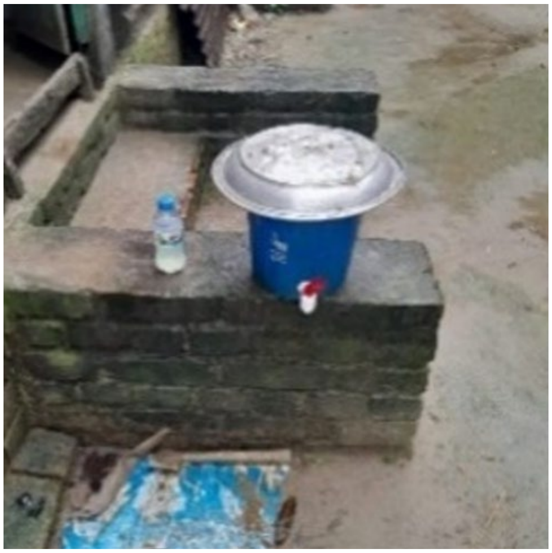
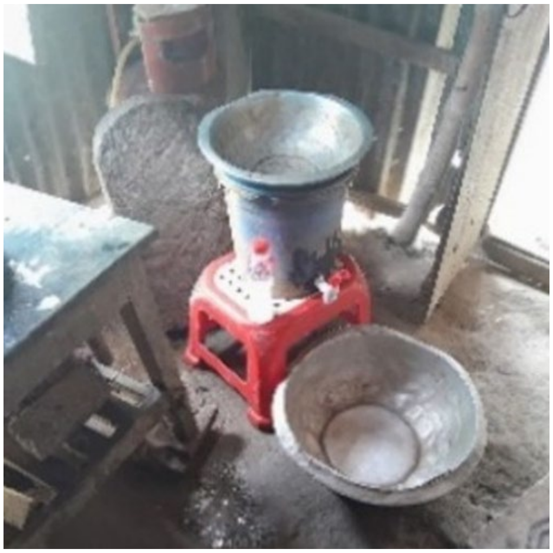

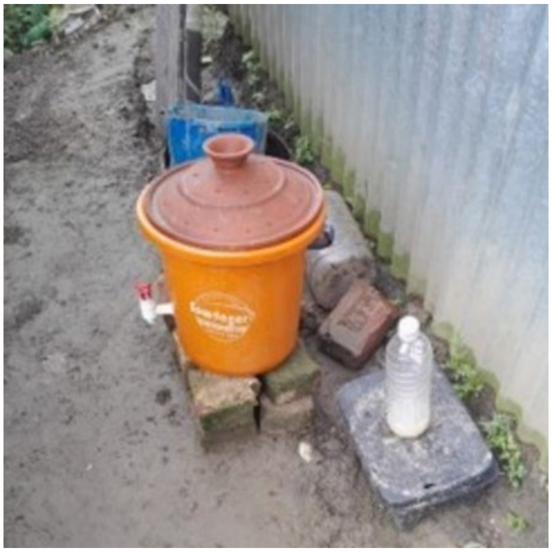

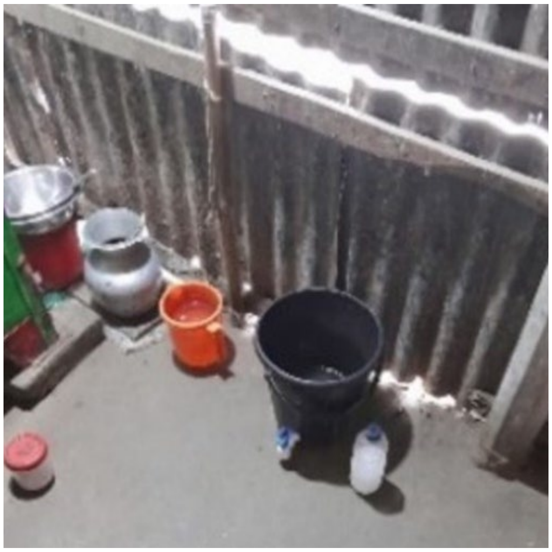

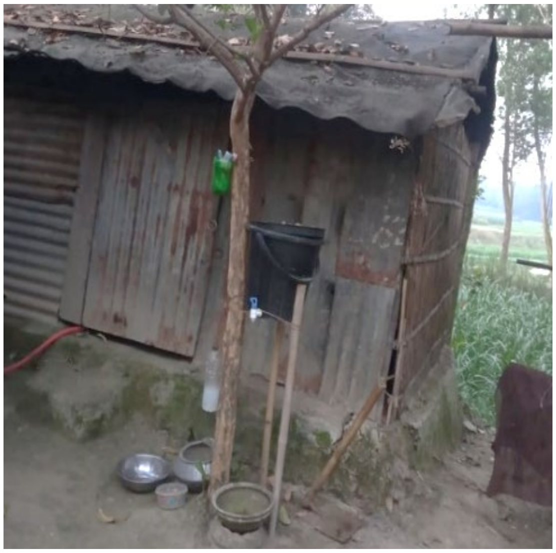

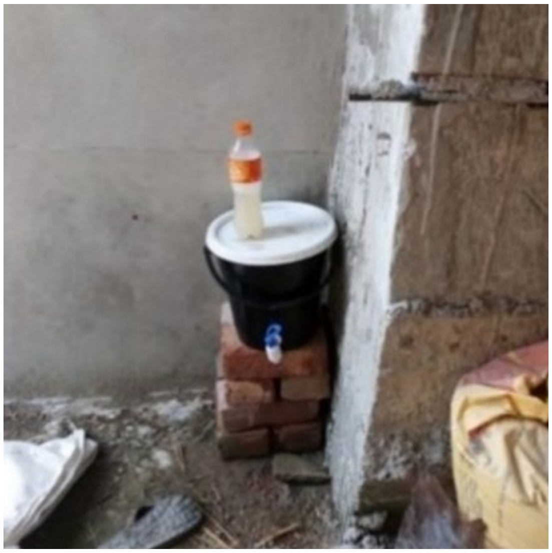

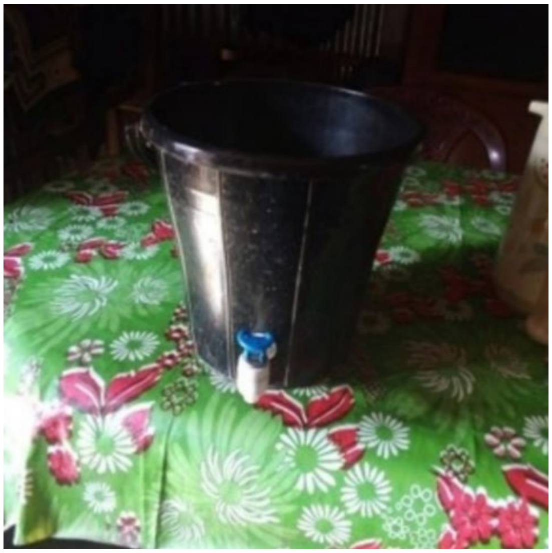

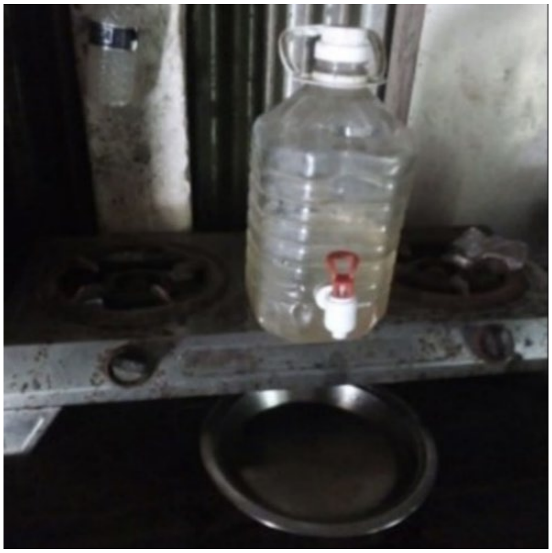

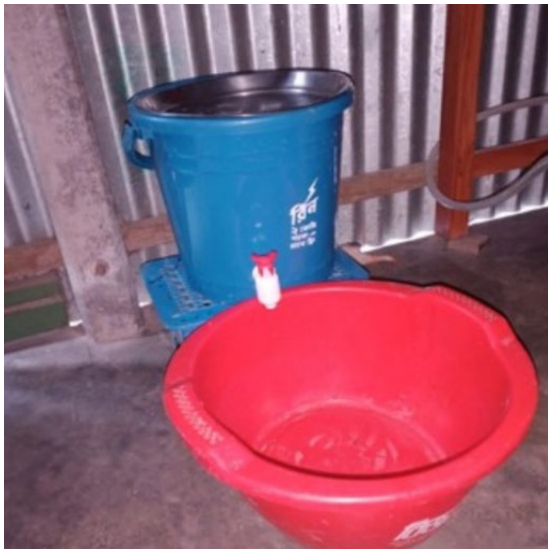

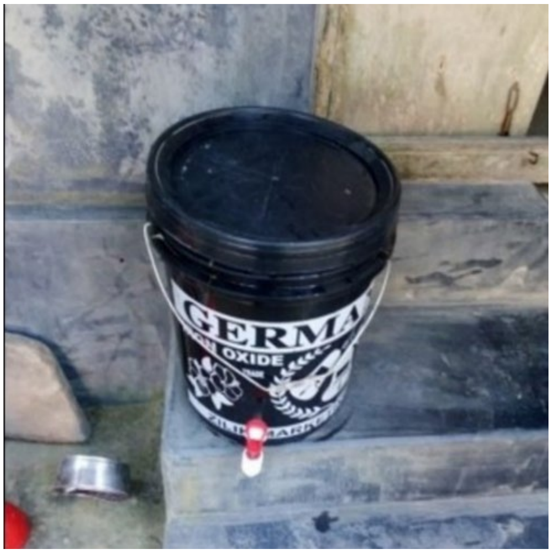
OXIDE

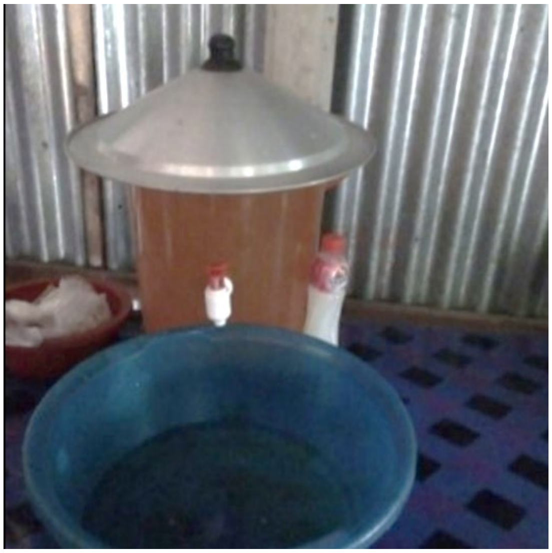
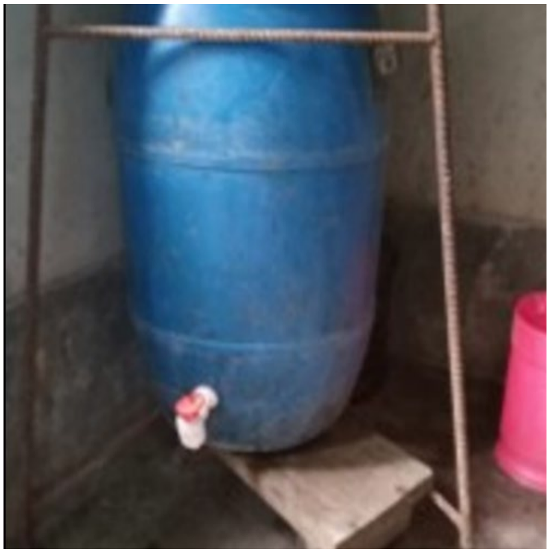
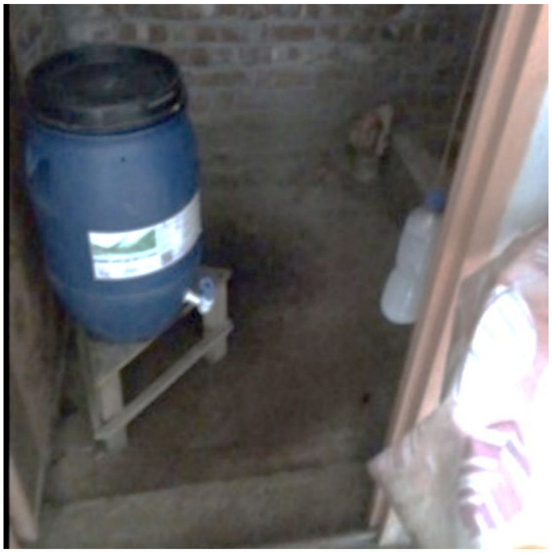
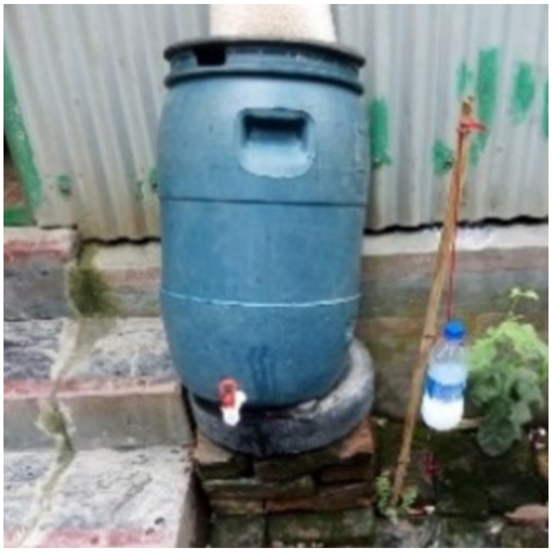

Bottle with a Hole at the Bottom Handwashing Station Design

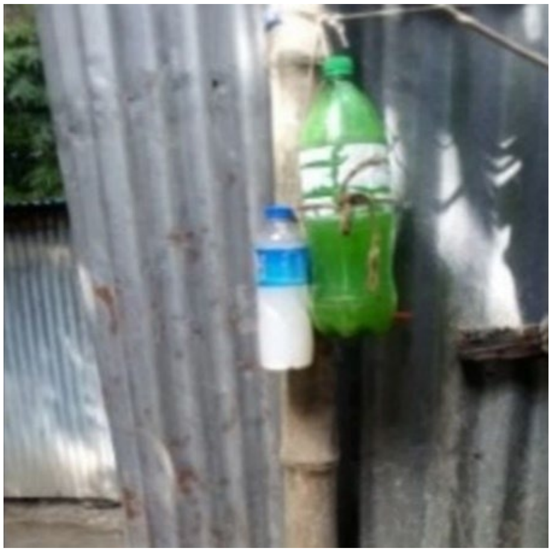

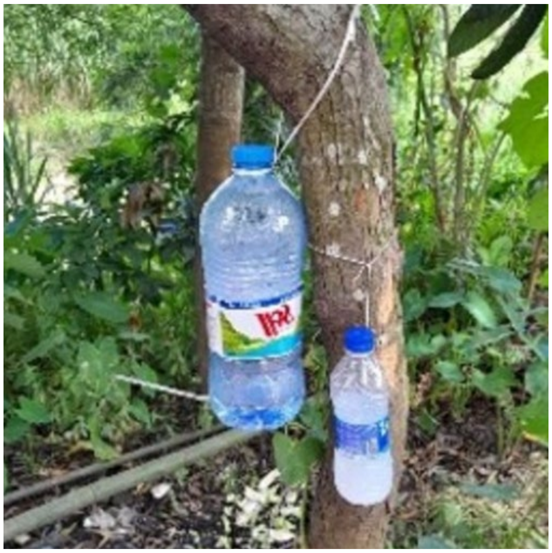

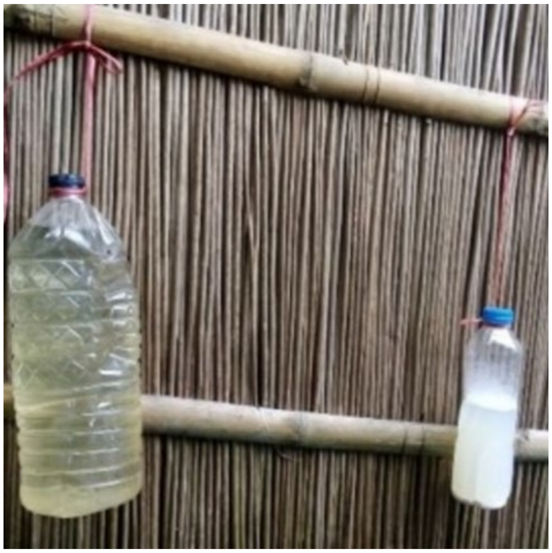

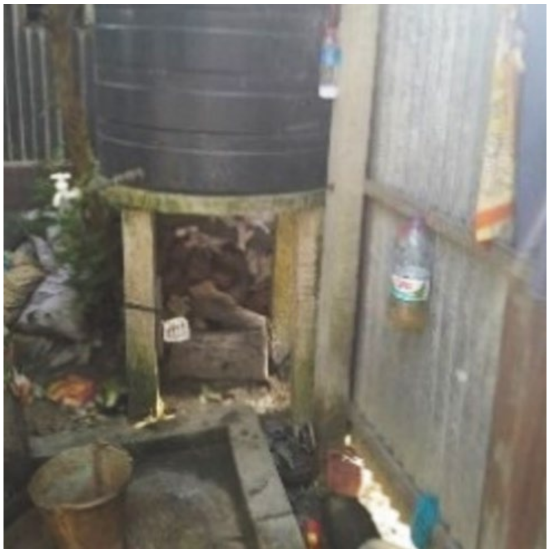

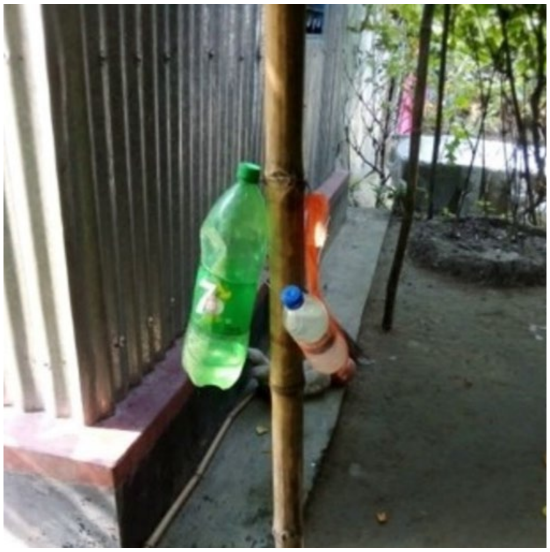

Bottle on Tubewell Handwashing Station Design

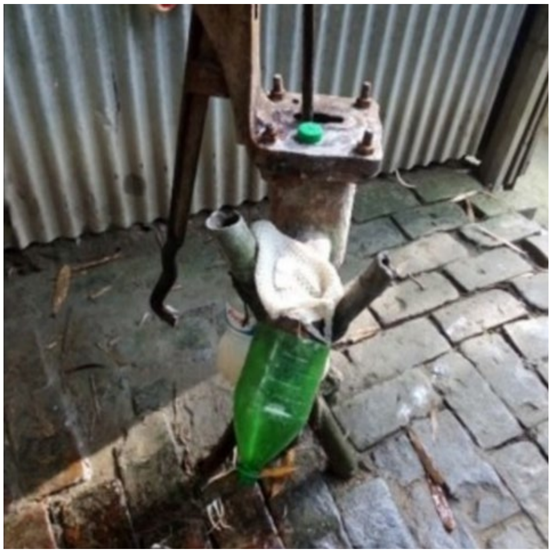

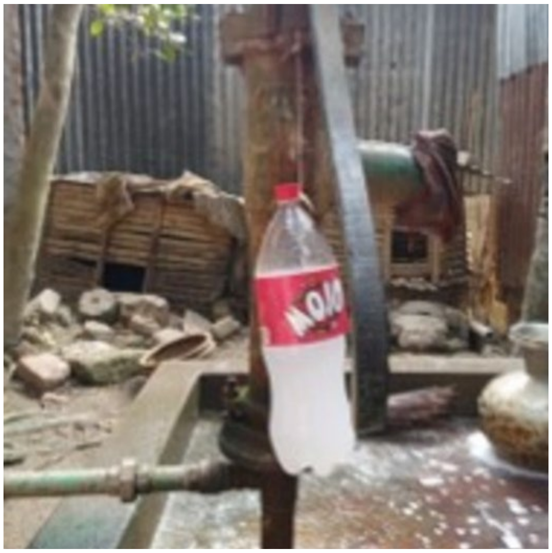

Supplementary Figure S3: Instructions on chlorine tablet dosing

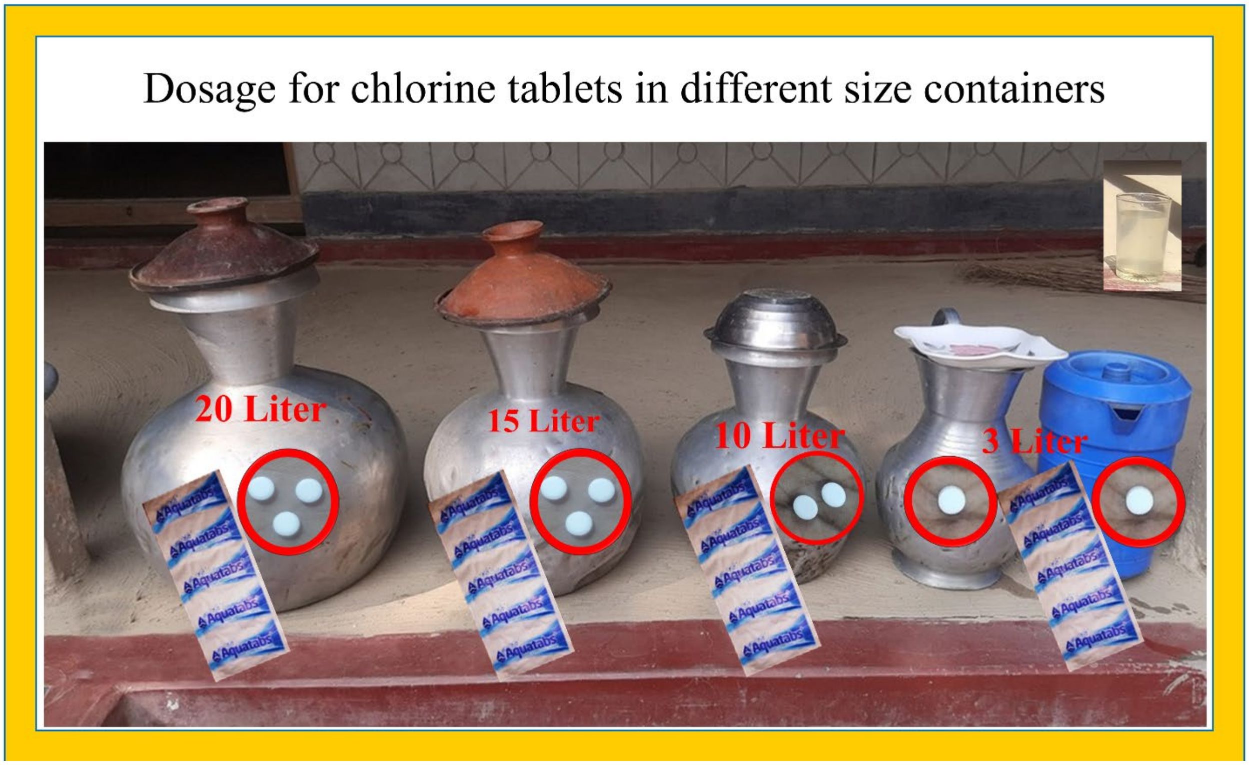

Supplementary Figure S4: Self-Made Iron Filter Designs Observed

Outside Appearance of Self-Made Iron Filters

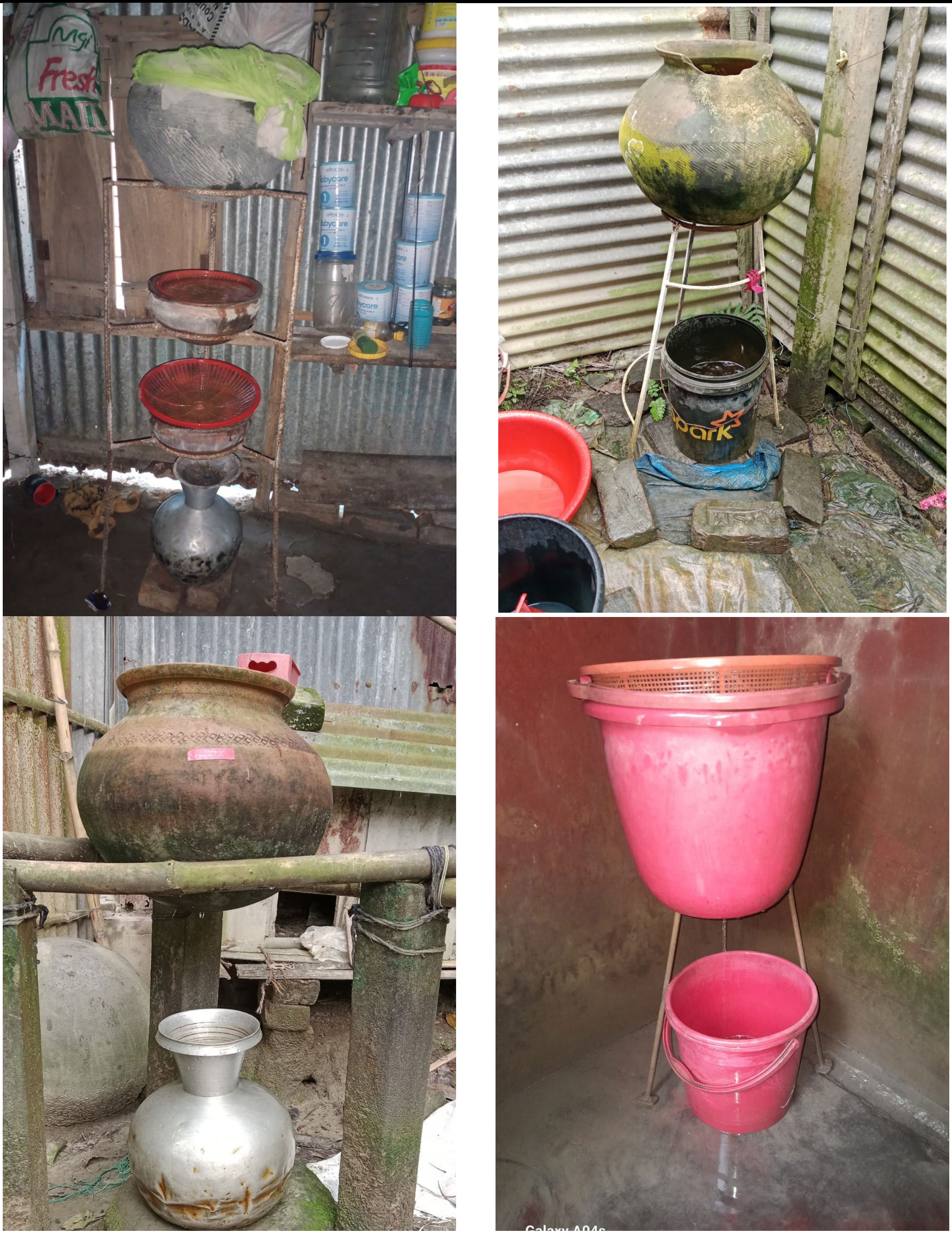

Media Inside Self-Made Iron Filters

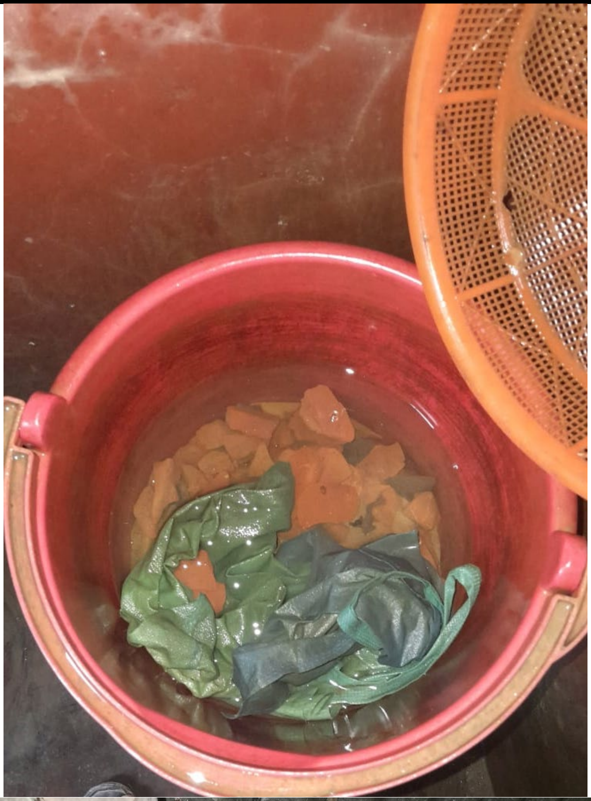

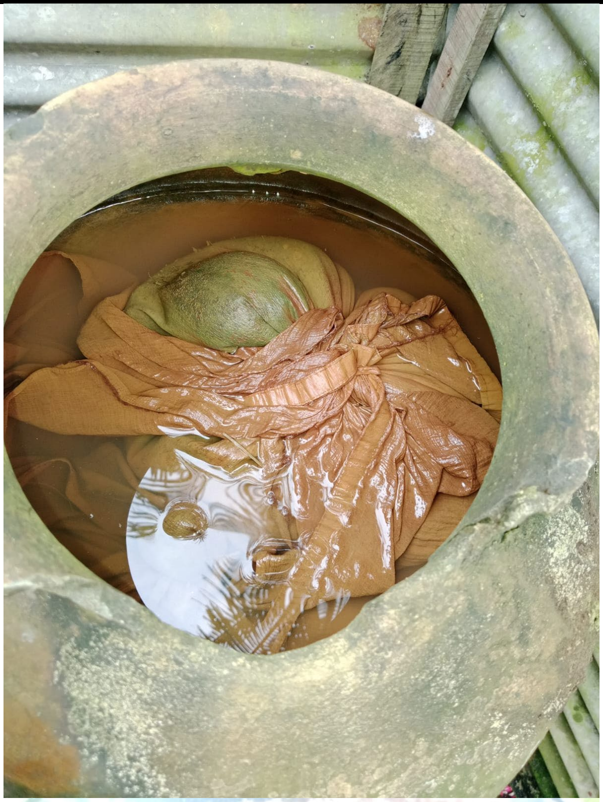

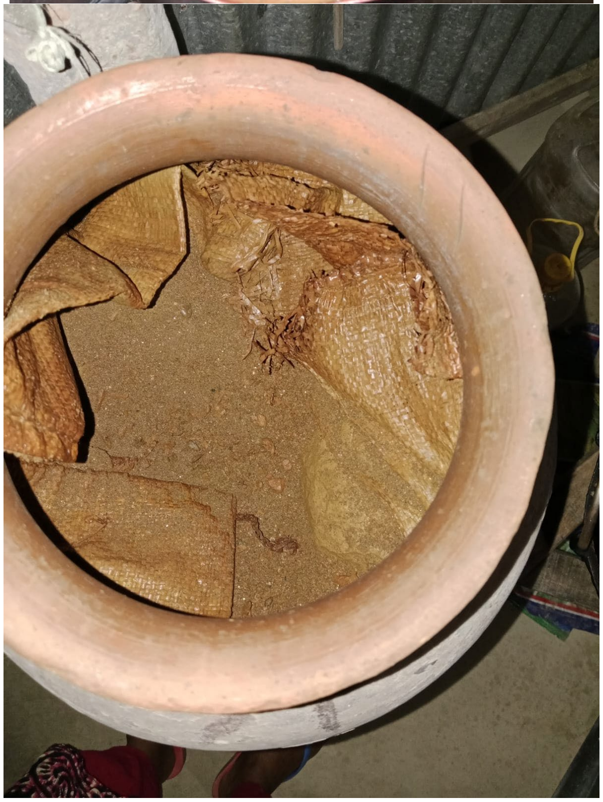

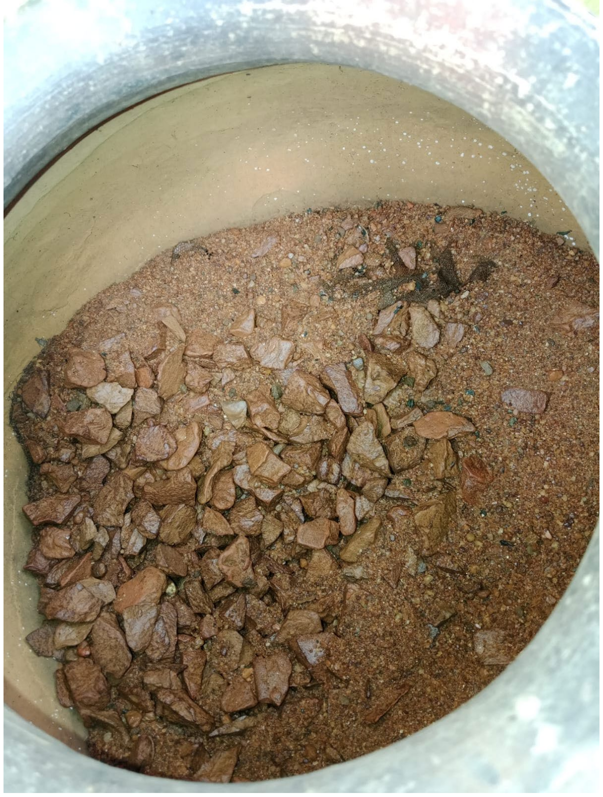

Supplementary Table S1. Semi-structured interviews and focus group discussions with study participants and implementers

Phase	Recipient SSI (Female)	Recipient SSI (Male)	Recipient FGD (Female)	Recipient FGD (Male)	Implementer SSI (Males and Females)	Total
Phase 1	6	3	3	1	0	13
Phase 2	0	4	5	5	0	14
Phase 3	0	0	0	0	0	0
Phase 4	2	1	0	0	0	3
Phase 5	2	3	0	0	4	9
Total	10	11	8	6	4	39

SSI= Semi-structured Interview

FGD= Focus Group Discussion

Supplementary Table S2. Overview of CHoBI7 Scale-Up intervention modifications over 5 pilot phases

Phase Introduced	Manuscript Section	Intervention Modification	Modification description
**Phase 1**	**3.1.1**	Initial Development of Modified Healthcare Facility CHoBI7 WASH Module	- Updated images in flipbook for the rural context,<br>- Added flipbook instructions on constructing a handwashing station using a bucket with a tap,<br>- Included "50 taka can buy happiness" video (a story of two families suffering from diarrhea demonstrating the difference between follower and non-followers of CHoBI7)<br>- Added health facility demonstration on water treatment using chlorine tablets using locally available water storage vessels<br>- Added a flipbook page on the high potential for tubewell drinking water contamination.
	**3.1.2**	Videos on WASH Behaviors	- Created 3-part video to promote targeted CHoBI7 program WASH behaviors<br>- Video Part 1: Testimonial of father of a young child that was a diarrhea patient explaining the importance of the CHoBI7 program<br>- Video Part 2: Video of of a young boy that became sick with diarrhea and was not able to play with his friends<br>- Video Part 3: video of a group of children reciting a CHoBI7 nursey rhyme explaining the key times to wash hands with soap and the importance of drinking chlorine treated water).
	**3.1.3**	Demonstrations of Handwashing Station Designs	- Handwashing station (bucket with tap) demonstration station set up in each healthcare facility<br>- Demonstrated how to make a hole in a plastic bucket to insert a tap during the healthcare facility visit<br>- Handwashing with soapy water demonstration in the healthcare facility
	**3.1.7**	Modified CHoBI7 Scale-up Program mHealth Component	- Content of the original CHoBI7 mHealth program component (i.e., mobile messages) was adapted to the rural context of the CHoBI7 Scale-Up program.
**Phase 2**	**3.1.8**	Mobile Message Emphasizing Preparing a Handwashing Station During 7-Day High-Risk Period	- Added automated voice call from Dr. Chobi asking households to prepare their handwashing station as soon as they come home from the health facility.
	**3.1.11**	Cue Card and Mobile Messages on Chlorine Tablet Dosing Instructions	- Cue card provided on the updated recommended chlorine dosing instructions based on the type of water storage container used.
	**3.1.13**	Mobile Messages Emphasizing that Chlorine Tablets Should be Used Beyond the 7-Day High Risk Period	- Included emphasis in mobile messages on the importance of chlorine tablets being used both during and after the7-day high risk period, and explaining that consuming chlorine tablets now doesn't prevent future diarrhea if use is stopped.
**Phase 3**	**3.1.4**	Introduction of a New Design of a Handwashing Station on a Tubewell	- New handwashing station design named "bottle on the tubewell" introduced that slows down the flow rate of water coming out of tubewell allowing user to wash hands without need for continuous water pumping.
	**3.1.5**	Introduction of a New Handwashing Station Design Using a Plastic Bottle with a Hole	- New handwashing station design named "bottle with a hole at the bottom" was introduced (large 2-liter bottle with a hole near bottom and a soapy water bottle hung from a tree or pole). The hole at the bottom of the bottle allowed for water to slowly come out for handwashing.
	**3.1.9**	Changing Timing of mHealth Message Delivery	- mHealth message delivery was moved 7:30 PM when most household members would be present,<br>- Automatically resent voice, IVR, and text messages that were not received by pilot participants within 24 hours using the Engagespark mobile platform.
	**3.1.12**	Mobile Messages Emphasizing That Iron Filters Do Not Remove Germs	- Added explaination to mHealth messages that iron filters do not remove diarrhea germs and recommended that households use their homemade iron filters first to remove the iron in water and then use a chlorine tablet to remove germs to make water safe for drinking.
	**3.1.14**	WASH Module (Flipbook) Clarifying That Arsenic- and Iron-Free Water Can Still Have Germs That Cause Diarrhea	It was emphasized in the flipbook module delivered in the healthcare facility that even tubewell water with no arsenic or iron could have germs that could cause diarrhea.
**Phase 5**	**3.1.6**	WASH Module (Flipbook) Page on Dos and Don'ts of Preparing a Handwashing Station	- Added one flipbook page on dos and don'ts of preparing a handwashing station using the pilot household photos<br>- Cue card was provided to pilot households on how to construct a handwashing station.
	**3.1.10**	Direct Mobile Phone Call by Health Promoters During 7-Day High-Risk Period	- Introduced two direct follow-up calls to households from health promoters on Day-2 and Day-6 of the high-risk period after the diarrhea patient was admitted to health facility for treatment to provide guidance on how to prepare a handwashing station and treat water with chlorine tablets<br>- testimonial videos reshared using WhatsApp.

Supplementary Table S3. CHoBI7 quantitative study findings

Follow-up Visit Timepoint		Handwashing station with water and any soap present		Observed hand-washing with soap		Free chlorine >0.2 mg/L	
		%	N	%	N	%	N
**Phase 1**	**Day 7**	13%	8	53%	15	0%	8
	**Month 1**	25%	8	24%	17	0%	8
	**Month 3**	0%	6	50%	12	0%	6
**Phase 2**	**Day 7**	13%	15	36%	39	7%	15
	**Month 1**	7%	14	31%	26	7%	14
	**Month 3**	13%	15	26%	39	7%	15
**Phase 3**	**Day 7**	45%	11	85%	29	55%	11
	**Month 1**	27%	11	36%	33	18%	11
	**Month 3**	0%	10	19%	27	45%	11
**Phase 4**	**Day 7**	60%	5	29%	14	40%	5
	**Month 1**	40%	5	33%	12	0%	5
	**Month 3**	20%	5	38%	8	67%	3
**Phase 5**	**Day 7**	50%	10	50%	30	56%	9
	**Month 1**	50%	10	36%	22	38%	8
	**Month 3**	50%	10	32%	25	63%	8
**Control (All Phases Combined)**	**Day 7**	0%	15	23%	35	20%	15
	**Month 1**	0%	15	6%	32	13%	15
	**Month 3**	0%	15	22%	32	47%	15
